# A segmentation method for lung nodule image sequences based on superpixels and density-based spatial clustering of applications with noise

**DOI:** 10.1371/journal.pone.0184290

**Published:** 2017-09-07

**Authors:** Wei Zhang, Xiaolong Zhang, Juanjuan Zhao, Yan Qiang, Qi Tian, Xiaoxian Tang

**Affiliations:** 1 College of Computer Science and Technology, Taiyuan University of Technology, Jinzhong, Shanxi, China; 2 College of Information Science and Technology, Pennsylvania State University, University Park, Pennsylvania, United States of America; 3 Department of Computer Science, University of Texas at San Antonio, San Antonio, Texas, United States of America; 4 Department of PET/CT center, Shanxi Provincial People's Hospital, Taiyuan, Shanxi, China; Beijing University of Technology, CHINA

## Abstract

The fast and accurate segmentation of lung nodule image sequences is the basis of subsequent processing and diagnostic analyses. However, previous research investigating nodule segmentation algorithms cannot entirely segment cavitary nodules, and the segmentation of juxta-vascular nodules is inaccurate and inefficient. To solve these problems, we propose a new method for the segmentation of lung nodule image sequences based on superpixels and density-based spatial clustering of applications with noise (DBSCAN). First, our method uses three-dimensional computed tomography image features of the average intensity projection combined with multi-scale dot enhancement for preprocessing. Hexagonal clustering and morphological optimized sequential linear iterative clustering (HMSLIC) for sequence image oversegmentation is then proposed to obtain superpixel blocks. The adaptive weight coefficient is then constructed to calculate the distance required between superpixels to achieve precise lung nodules positioning and to obtain the subsequent clustering starting block. Moreover, by fitting the distance and detecting the change in slope, an accurate clustering threshold is obtained. Thereafter, a fast DBSCAN superpixel sequence clustering algorithm, which is optimized by the strategy of only clustering the lung nodules and adaptive threshold, is then used to obtain lung nodule mask sequences. Finally, the lung nodule image sequences are obtained. The experimental results show that our method rapidly, completely and accurately segments various types of lung nodule image sequences.

## Introduction

Lung cancer is one of the most malignant tumors with the highest morbidity and mortality [1, 2]; therefore, it is very important to detect lung nodules as early as possible and make an early diagnosis. X-ray computed tomography (CT) [3, 4] plays an important role in early lung cancer detection and tumor diagnosis because of the precise structural features of these tumors. To improve the detection rate of lung nodules, a thin-section CT image is often used, but this method also increases the amount of data and the noise in the images, leading to a poor segmentation efficiency and the introduction of noise. In addition, the probability of malignancy for cavitary nodules and juxta-vascular nodules is very large; thus, the accurate segmentation of these two types of nodules has great significance for improving patient survival. Therefore, in the field of modern medicine, there is an urgent need for a method that can accurately segment lung nodule image sequences while satisfying the clinical requirement for speed.

In the computer-aided diagnosis system [5, 6], the segmentation effect of lung nodules has a direct impact on the accuracy of the subsequent feature extraction and diagnosis. Therefore, in recent years, research examining segmentation methods for lung nodule images has attracted increasing attention.

Sun S et al. [7] proposed the k-means (KM) algorithm using flow entropy and geodesic distance to segment nodules, which has a better segmentation effect. However, with an increase in the nodule radius, the longer the algorithm execution time, the more the efficiency declines. Chen K et al. [8] used the fuzzy speed function to optimize the active contour model to segment lung nodules, which can effectively solve the problem of segmentation boundary leakage of the traditional active contour model, but the algorithm performance depends largely on the choice of the initial position. Zinoveva O et al. [9] proposed a pixel classification method based on texture features. Achievement of the classifier provides an effective distinction between the blood vessels and nodules, which can provide better segmentation results for lung nodules. However, algorithms based on pixels are inefficient. Xu N et al. [10] proposed a method for automatic lung nodules segmentation based on dynamic programming and expectation maximization (EM) classification. First, the method used the shape constraint model to segment the object boundary and calculate the optimal object boundary by dynamic programming. Then, the use of an EM classification algorithm to remove calcification provided a general treatment effect for cavitary nodules and juxta-vascular nodules. Wei Y et al. [11] proposed a mean-shift clustering method combined with multi-scale Hessian dot filtering, which uses a multi-scale Hessian matrix to enhance nodules and to restrain the linear structure of the trachea and blood vessels. Subsequently, a mean-shift clustering kernel function was designed by calculating the shape filtering value of the Hessian matrix, gray value and spatial position of three feature information types. Finally, segmentation of the suspected regions of interest (ROIs) of lung nodules was achieved. Sivakumar S et al. [12] proposed an unsupervised method for lung nodule segmentation. Based on the study of several unsupervised segmentation models, a probabilistic fuzzy clustering algorithm is proposed. Compared with fuzzy c-means (FCM), possibilistic c-means and fuzzy possibilistic c-means, this algorithm can improve the speed and accuracy of segmentation. Kong Y et al. [13] proposed new unsupervised and supervised information theoretic discriminative algorithms to segment brain MRI images. First, the simple linear iterative clustering algorithm is employed to generative three-dimensional (3D) supervoxel samples. The intensity, texture and shape features are then extracted to describe each supervoxel. A discriminative learning method based on theoretical information is then utilized to manage tissue heterogeneity and feature redundancy. Compared with KM, MI, MRF and WPNN, the proposed algorithm indeed improves the segmentation performance for brain MRI datasets. Deng Y et al. [14] proposed a fused fuzzy deep neural network for data classification. First, the fuzzy membership function is used to calculate the fuzzy degree of all input nodes. Then, neural learning is exploited to transform the input as a high-level representation. Moreover, the multi-model neural network structure is employed to fuse neural and fuzzy representations. Finally, the soft-max function is applied to classify the data. Compared with NLFS, MRF, ITS, DNN, SCFDNN and SFDNN, the joint learning network framework produces the best segmentation results for IBSR and BrainWeb brain datasets.

Although these algorithms for the segmentation of solid nodules are very effective, the following issues are raised. (1) The segmentation of lung nodule image sequences is ineffective without reducing accuracy. (2) In cavitary nodules, there are large differences in the gray values between the cavity and the remaining area; as such, the cavity is easily regarded as part of the lung parenchyma, which leads to incomplete nodule segmentation [15]. (3) The gray value of juxta-vascular nodules is very close to that of blood vessels, facilitating the merging of the blood vessels and nodules and thus ineffectively separating them. (4) There is increased noise, leading to the introduction of noise and various tissue structures into the lung nodule segmentation.

Therefore, to address these problems in this paper, we investigated many algorithms. We propose a segmentation method for lung nodule image sequences based on superpixels and density-based spatial clustering of applications with noise (DBSCAN). This method not only significantly reduces the effects of noise but also completely incorporates the features of color, spatial location, blood vessels and nodule type in the CT images. Compared with other existing methods mentioned in the literature, our method is more efficient and accurate for segmented cavitary nodules and juxta-vascular nodules.

## Materials and methods

### Materials

#### Ethics statement

This study was approved by the institutional review board of the Shanxi Provincial People’s Hospital. The study was conducted in accordance with the hospital’s ethical requirements. Informed consent was obtained from all patients prior to study inclusion.

#### Datasets

The datasets were obtained from two different databases: Shanxi Provincial People's Hospital, which uses PHILPS 256 CT (Philips iCT) layer speed equipment (240 mA, 120 kV, slice thickness of 1.5 mm), and the Lung Image Database Consortium (LIDC) [16] (40~422 mAs, 120~140 kVp, slice thickness ranging from 1 mm to 3 mm). All data can be obtained at https://figshare.com/s/7564b0623360d92577a2. In the experiment, the datasets contained 1458 lung CT sequence images collected from 120 clinical cases, and the size of each CT image was 512×512. The experimental datasets were also classified by experienced imaging physicians. Nodules were categorized as solitary pulmonary nodules, cavitary nodules or juxta-vascular nodules, and approximately 40 cases were included for each nodule type.

### Proposed method

**Fig 1:**
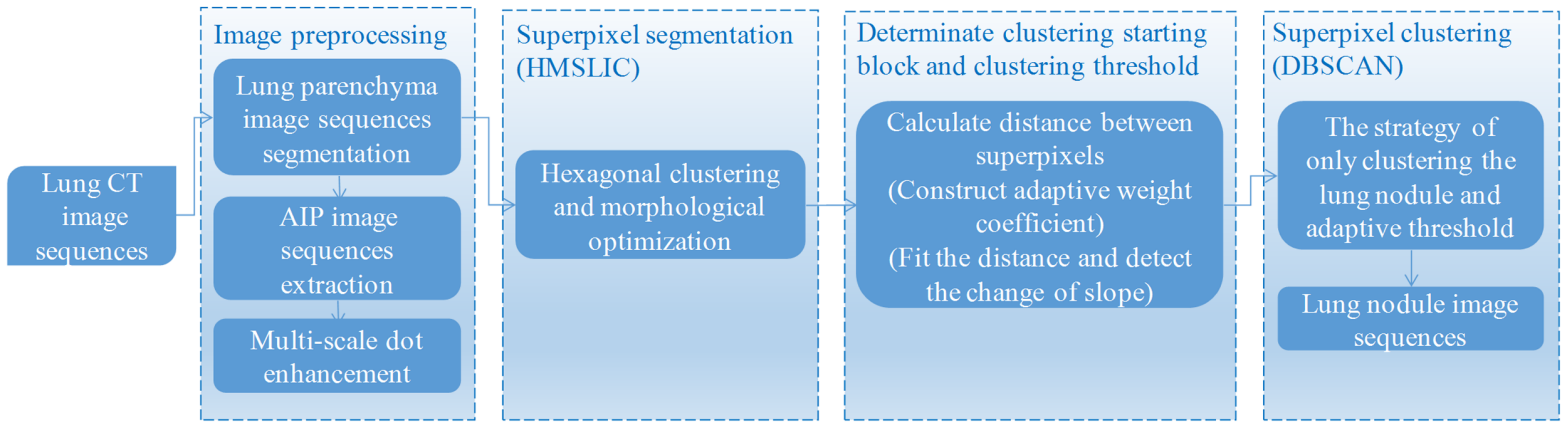
Diagram of the lung nodule image sequence segmentation method.

This paper focuses on the lung sequence images of solitary pulmonary nodules, cavitary nodules and juxta-vascular nodules. We present an efficient and accurate segmentation method. A diagram of the lung nodule image sequence segmentation method is shown in Fig 1.

#### Pretreatment

To better eliminate the influence of many bright regions on the segmentation of lung nodules, the following features were included in the pretreatment work.

**1. Lung parenchyma image sequence segmentation:** In this section, we use the lung parenchyma segmentation method [17] proposed by the project group to segment the lung parenchyma image sequences. The method is divided into four stages. First, we use the location features of the lung parenchyma images to obtain lung CT ROI image sequences. Second, a gradient and sequential linear iterative clustering algorithm is utilized to segment the ROI image sequences. Third, the self-generating neural forest (SGNF), which is optimized by a genetic algorithm, is proposed for the superpixel clustering. Fourth, the gray and geometric features of the superpixels are used to identify the lung parenchyma. Finally, lung parenchyma sequence images are obtained.

**2. Average intensity projection (AIP) image sequence extraction:** Considering that this method can be used to weaken the gray of blood vessels and recover the shape of blood vessels in lung CT images, the method can also effectively eliminate the influence of the long strip and blob-like blood vessels on lung nodules segmentation. Therefore, in this paper, the AIP [18] is used for the sparse sampling of original CT sequence images by calculating the mean gray of the points on each ray of the continuous CT images. The AIP is defined as follows in this paper:

$$AIP(x,y)=\frac{1}{S_{r}}\sum_{i=1}^{S_{r}}G_{i}(x,y),1\leq x\leq 512,1\leq y\leq 512$$ (1)

where *AIP*(*x*, *y*) is the gray value at point (*x*, *y*) on the *AIP* image, *S*_*r*_ is the number of slices for the *AIP* reconstruction areas, and *G*_*i*_(*x*, *y*) is the gray value at point (*x*, *y*) on the i-th CT image. The *S*_*r*_ is defined as

$$S_{r}=T_{r}/t$$ (2)

where *T*_*r*_ is the section thickness of the AIP reconstruction and *t* is the slice thickness of the original CT image.

**Fig 2:**
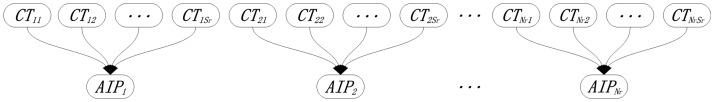
Mapping relationship between CT sequence images and AIP sequence images.

The mapping relationship between the lung CT sequence images and AIP sequence images is shown in Fig 2, and *N*_*r*_ is the total number of AIP reconstruction image sequences.

**3. Multi-scale dot enhancement:** For two-dimensional (2D) images, the Hessian matrix is defined as

$$\text{H}=\left[\begin{array}{cc} I_{xx} & I_{xy}\\ I_{yx} & I_{yy} \end{array}\right]$$ (3)

The set parameters *λ*_1_ and *λ*_2_ are two eigenvalues of the Hessian matrix. The linear structure satisfies the condition | *λ*_1_ |≈0, | *λ*_2_ |>> 0, and the circular structure satisfies | *λ*_1_ |≈|*λ*_2_|>> 0. Thus, Frangi [19] proposed the vessel similarity function to enhance the blood vessels. However, we propose the dot similarity function based on the circular features of lung nodules to achieve dot enhancement. The function is defined as

$$d_{o}(\lambda )=\{\begin{array}{cc} 0 & \lambda_{2}>0\\ \left(1-\text{exp}(-\frac{R_{A}^{2}}{2\alpha^{2}}))(1-\text{exp}(-\frac{R_{B}^{2}}{2\beta^{2}})\right) & Other \end{array}$$ (4)

In Eq (4),$R_{A}=\frac{\left|\lambda_{1}\right|}{\left|\lambda_{2}\right|},\;R_{B}=\sqrt{\lambda_{1}^{2}+\lambda_{2}^{2}}$

where *α*, *β* is used to set the weights of *R*_*A*_ and *R*_*B*_, and the value of *β* is generally affected by the scale range of the gray image, with a smaller value resulting in a greater response to the change in gray. When it becomes a linear structure, *R*_*A*_ = 0, and *d*_*o*_(*λ*) = 0. Therefore, *R*_*A*_ can be used to distinguish the circular structure and the linear structure. When it becomes a noise point, the eigenvalues *λ*_1_ and *λ*_2_ are very small and *R*_*B*_ = 0, and *d*_*o*_(*λ*) = 0. Therefore, *R*_*B*_ can be utilized to distinguish the circular structure and the noise point.

The combination of the Hessian matrix and Gaussian function is utilized to achieve multi-scale fusion. The Gaussian function is defined as

$$\text{G}(X,\sigma )=\text{exp}(-{\frac{\left||X|\right|}{2\sigma^{2}}}^{2})$$ (5)

where *σ* is the standard deviation of the Gaussian function, which is the spatial scaling factor. Based on mathematical knowledge, because 95% of the weights of the Gaussian function are included between [-2σ, 2σ], the diameter of the circular structure is approximately 4σ. When σ is best matched with the actual scale of the circular structure, the response of the dot enhancement is greatest. Thus, the experimental σ is defined as follows:

$$\sigma_{i}=\sigma_{\text{min}}+i\times \frac{\sigma_{\text{max}}-\sigma_{\text{min}}}{N-1},i=0,1,\cdot \cdot \cdot ,N-1$$ (6)

In Eq (6), *σ*_min_ = *d*_min_/4, *σ*_max_ = *d*_max_/4

where *N* is the number of scales, *d*_*min*_ is the minimum diameter of the lung nodule images, and *d*_*max*_ is the maximum diameter of the lung nodule images.

Finally, the method selects the point of maximum response of each scale as the output by continuous iterative *σ* to obtain CT images after dot enhancement.

#### Hexagonal clustering and morphological optimized sequential linear iterative clustering (HMSLIC)

In 2003, RenXiao-feng et al. proposed the theory of superpixels [20]. A superpixel is a collection of adjacent pixels with similar brightness, color and texture. Compared with previous pixel-based image processing methods, the combination of superpixels with similar pixels can greatly reduce the complexity of subsequent image processing tasks and improve the operation speed of segmentation algorithms.

In 2009, Achanta et al. proposed a simple linear iterative clustering (SLIC) [21, 22] algorithm based on the original superpixel segmentation method; this algorithm uses a five-dimensional feature vector **[*L*, *a*, *b*, *x*, *y*]**^**T**^ to calculate **[*x*,*y*]**, representing the pixel position, and **[*L*, *a*, *b*]**, representing the three-color information ***L*, *a*, *b*** of the ***Lab*** color space. Considering the similarity of the color and location features, superpixels with a compact structure and strong homogeneity are generated.

In this paper, we propose an improved superpixel segmentation algorithm, named hexagonal clustering and morphological optimize sequential linear iterative clustering algorithm (HMSLIC), specially for the lung CT images, according to the circular and size features of the lung nodules.

Moreover, HMSLIC segmentation is only focused on the lung parenchyma. For areas with a gray level of zero, only a simple hexagonal grid is needed, rather than a cluster center update operation. This procedure significantly reduces the time complexity of the image sequence segmentation. The **HMSLIC** algorithm is shown in Table 1.

**Table 1:** HMSLIC algorithm.

*Algorithm 1 HMSLIC*
1: Initialize cluster center ***C***_***k***_ = **[*L***_***k***_, ***a***_***k***_, ***b***_***k***_, ***x***_***k***_, ***y***_***k***_**]**^**T**^ in the hexagonal grid, pixels label matrix ***l***, distance matrix ***D*** from pixels to cluster centers, hexagonal grid spacing ***S*** and radius ***r*** of circular structure element 2: Move initial cluster center to the smallest gradient position in the hexagonal region. 3: ***Repeat*** 4: ***while*** residual error ***>*** = threshold ***E do*** 5: ***for*** each cluster center ***C***_***k***_ ***do*** 6: Obtain sub images (use **[*x***_***k***_, ***y***_***k***_**]** as the center, 2*S as the side) containing cluster centers. Calculate the distance between each pixel in the sub image and cluster center. If the distance is less than the previous value, then update its ***l*** and ***D***. 7: ***end for*** 8: Calculate the mean of ***L***, ***a***, ***b***, ***x*** and ***y*** of each superpixel to update the cluster center. Recalculate the residual error, go to the third step, and continue the execution. 9: ***end while*** 10: Obtain all non-connected regions of the CT image and then perform the open operation of the circular structure element radius ***r***. The final result is subtracted from the original CT image denoted as ***mask***. 11: Perform distance transform on ***~mask***, and assign each small region to the nearest superpixel. 12: Compute the superpixel adjacency matrix for subsequent operation. 13: ***Until*** all CT image sequences are segmented. 14: Execute connection operations and sequential output.

In the HMSLIC algorithm, the size of the input image is assumed as *rows* * *cols*, which is divided into *K* superpixels. Thus, the hexagonal grid spacing $S=\sqrt{2*rows*cols/K*\sqrt{3}}$, and the number of superpixels in each column is *blockCols* = [*cols*/*S* − 0.5]. The cluster centers are used as starting points to search for similar pixels.

The algorithm measures the similarity between pixels by calculating the distance matrix ***D*** from each pixel in each sub image to the cluster center. The distance between pixels includes the color distance ***d***_***c***_ and space distance ***d***_***s***_, and thereby the distance matrix is defined as

$$d_{s}={\left(d_{x}-x_{k}\right)}^{2}+{\left(d_{y}-y_{k}\right)}^{2}$$ (7)

$$d_{c}={\left(d_{l}-L_{k}\right)}^{2}+{\left(d_{a}-a_{k}\right)}^{2}+{\left(d_{b}-b_{k}\right)}^{2}$$ (8)

$$D=\sqrt{d_{c}+\frac{d_{s}*m^{2}}{S_{cluster}{}^{2}}}$$ (9)

where *S*_*cluster*_ = [*cols*/(*blockCols*+0.5)], and *S*_*cluster*_ represents the spacing between cluster centers. ***d***_***x***_ represents the space distance matrix from the *x* coordinates of all pixels in the sub image corresponding to the k-th clustering center to the *x* coordinates of the clustering center. Similarly, ***d***_***l***_ represents the color distance matrix from the *L* component of all pixels in the sub image corresponding to the k-th clustering center to the *L* component of the clustering center. ***m*** represents the weight coefficient between the space distance and the color distance, which is generally between 1 and 40. The greater the value, the more compact but the less sensitive the segmented superpixels become to changes in color. The smaller the value, the more sensitive the segmented superpixels become to changes in color, although the shape of the superpixels becomes more irregular.

The open operation of structural element B on A, denoted as A∘B, is defined as

A∘B=(AΘB)⊕B (10)

That is, the structural elements are used to perform the erosion operation [23] on A; then, the same structural element is used to perform the dilation operation [23].

The improved algorithm is mainly reflected in two aspects. First, the HMSLIC algorithm uses a hexagonal method that is approximately consistent with the circular shape features of lung nodules to achieve clustering. Thus, the process of pixel clustering will make up for the disadvantage that the clustering effect of the quadrilateral differs in different directions. By performing the HMSLIC algorithm, the segmented superpixels are more regular and the edge information is well preserved. Second, the HMSLIC algorithm uses morphology to combine bright superpixels, which are less than the fixed area to the nearest superpixels. It further eliminates the influence of bright blob-like blood vessels. Additionally, after the merger, the number of superpixels is greatly reduced, which greatly reduces the complexity of the subsequent calculation of distance.

### Superpixel sequence clustering based on the improved DBSCAN algorithm

This paper uses a fast DBSCAN superpixel sequence clustering algorithm, which is optimized by the strategy of only clustering the lung nodules and adaptive threshold to achieve superpixel sequence clustering.

**1. DBSCAN:** DBSCAN [24, 25] is a spatial clustering algorithm based on density. The algorithm divides the areas with sufficient density into clusters and identifies clusters with an arbitrary shape in spatial databases with noise. It defines clusters as the largest collection of density-connected objects. In addition, it displays characteristics of insensitivity to noise data and the order of samples in the database.

Some concepts of the DBSCAN algorithm include the following:

*E*-neighborhood: The neighborhood within the radius (*E*) of the given object.
Core object: The *E*-neighborhood of an object contains at least a minimum number of objects (*MinPts*).
Directly density-reachable: For the object set *D*, if *q* is in the *E*-neighborhood of *p* and *p* is the core object, then object *q* is directly density-reachable from *p*.
Density-reachable: For the object set *D*, given a string of objects (*p*_1_, *p*_2_…*p*_*n*_, *p* = *p*_1_, *q* = *p*_*n*_), if object *p*_*i*_ is directly density-reachable from *p*_*i*−1_, then object *q* is density-reachable from *p*.
Density-connected: If objects *p* and *q* are density-reachable from object *o* in the object collection *D*, then *p* and *q* are density-connected.

The algorithm requires two parameters: the scan radius (*E*) and a minimum number (*MinPts*) of *E*-neighborhoods for the core object. The **DBSCAN** algorithm is shown in Table 2.

**Table 2:** DBSCAN algorithm.

*Algorithm 2 DBSCAN*
1: Initialize scan radius ***E***, the minimum number (***MinPts***) of *E*-neighborhoods of the core object. 2: ***Repeat*** 3: Extract an object ***p*** that has not been marked as visited from the database. 4: ***if p*** is the core object 5: ***then p*** and objects in the ***E***-neighborhood form a cluster, marking ***p*** as visited. The same method is then used to address all the objects that are not marked as visited in the neighborhood, achieving expansion of the cluster 6: ***else p*** is noise (non-core object) and ends this loop, followed by a search for the next object in the database. 7: ***end if*** 8: ***Until*** all objects in the database are processed (all objects are classified into a cluster or labeled noise).

**2. Adaptive clustering initiating the block and clustering threshold setting:** By calculating the dot similarity function and analyzing the features of the CT images after pretreatment, we present a method for quickly locating the position of lung nodules. Similarly, the method uses five-dimensional feature vectors **[*L***_***m***_, ***a***_***m***_, ***b***_***m***_, ***x***_***m***_, ***y***_***m***_**]**^**T**^ to calculate the distance between superpixels. The distance is defined as

$$d_{Lab}(b_{i},b_{j})={\left(L_{mi}-L_{mj}\right)}^{2}+{\left(a_{mi}-a_{mj}\right)}^{2}+{\left(b_{mi}-b_{mj}\right)}^{2}$$ (11)

$$d_{xy}(b_{i},b_{j})={\left(x_{mi}-x_{mj}\right)}^{2}+{\left(y_{mi}-y_{mj}\right)}^{2}$$ (12)

$$d(b_{i},b_{j})=\sqrt{d_{Lab}(b_{i},b_{j})+w^{2}*d_{xy}(b_{i},b_{j})}$$ (13)

$$w=mean(d_{o}{\left(\lambda \right)}_{i})$$ (14)

where *L*_m_, *a*_*m*_, *b*_*m*_ is the mean of the three color feature components. *x*_*m*_, *y*_m_ is the center location of the superpixel. *d*_*Lab*_(*b*_*i*_, *b*_*j*_) is the color feature similarity distance of superpixels *i* and *j*. *d*_*xy*_(*b*_*i*_, *b*_*j*_) is the space location feature similarity distance of superpixels *i* and *j*. *d*(*b*_*i*_, *b*_*j*_) is the distance between superpixels after the superposition of weight. *w* is the adaptive weight coefficient of the space feature distance. *d*_*o*_(*λ*)_*i*_ is the mean of the dot similarity of all pixels in the i-th superpixel.

In this paper, the value of the adaptive weight coefficient (*w*) is in the range of [0,1]. When a superpixel block is in the area of a blood vessel, the value of *w* is very small. In contrast, when a superpixel block is in the area of a nodule, the value of *w* is relatively large. Therefore, using this method to calculate the sum of the distance of each superpixel, which has the function of widening the difference in the sum of the distance between blood vessels and nodules, can better distinguish blood vessels and nodules.

**Fig 3:**
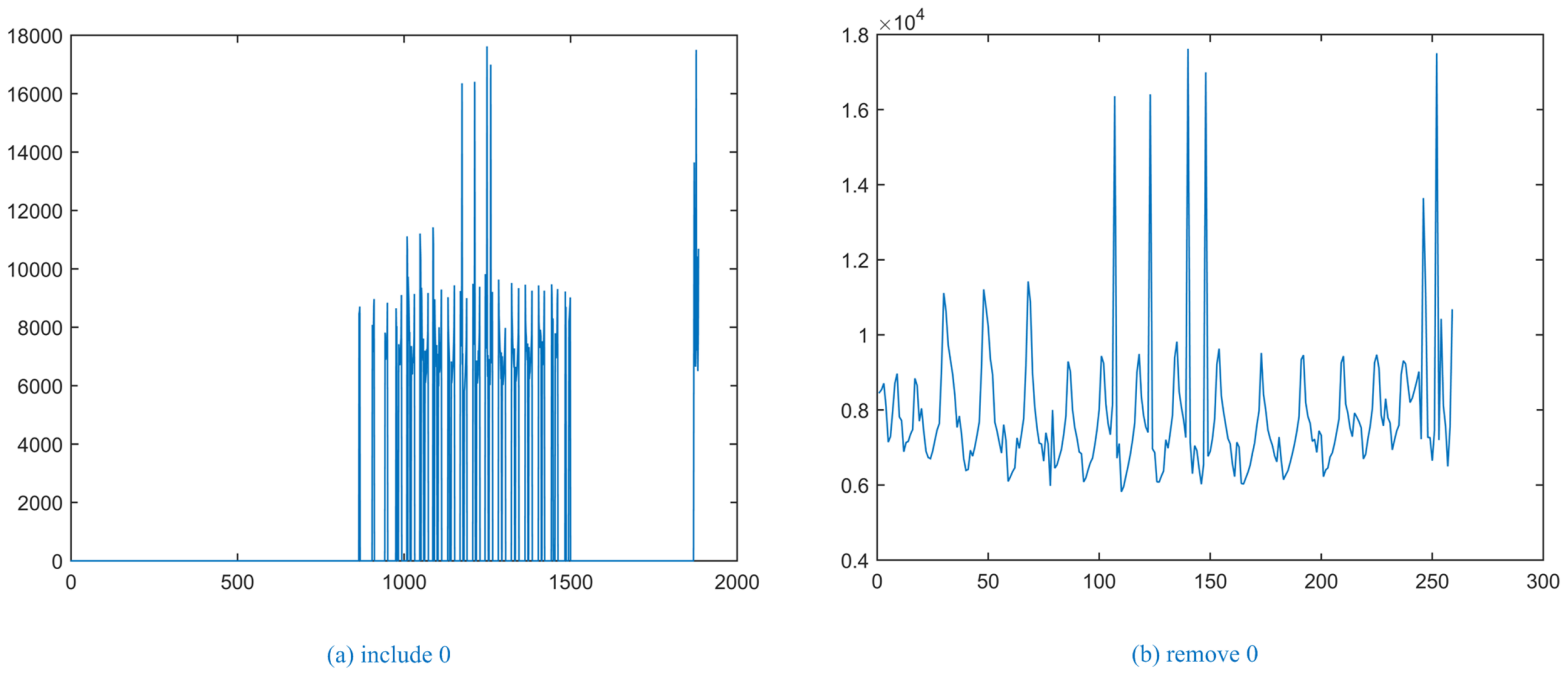
The sum of the distance between superpixel blocks.

Moreover, with the increased number of superpixels, the time complexity of the computation of distance is also greatly increased. Fig 3 shows that it is meaningless to calculate the distance in the area, excluding the lung parenchyma (as shown in Fig 3(a)). Therefore, in this paper, the matrix of the sum of the distance uses a sparse matrix and any distance between the superpixels with *L* = 0 set directly to zero and no longer calculated (as shown in Fig 3(b)).

As shown in Fig 3(a), the superpixel label for the maximum sum of the distance is 1249, which corresponds to the location of a lung nodule, resulting in its precise positioning and use as a subsequent clustering starting block.

Due to differences in the gray level between CT images, if only manual debugging is used to obtain the best threshold, then the process will become very time consuming when the debug spacing is too small, and threshold errors will arise when the debug spacing is too large.

Therefore, we present a fast and accurate method for determining the clustering threshold of DBSCAN. The method considers the gray differences between CT images and the features of various types of lung nodule images. The clustering threshold can then be adjusted automatically according to specific CT images and the nodular type.

**Fig 4:**
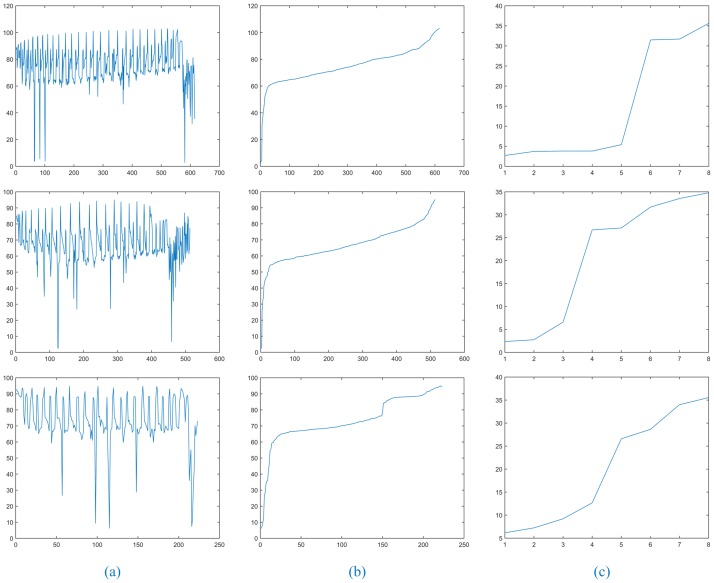
The relationship of the distance between index_max and other superpixels.

In Fig 4, the first line shows the results for a solitary pulmonary nodule image, the second line shows the results for a cavitary nodule image, and the third line shows the results for a juxta-vascular nodule image. In the experiment, after recording the superpixel label of the maximum sum of the distance as *index_max*, extracting data for the *index_max* column from the distance matrix, and removing 0 items, the results are as shown in Fig 4(a). For the same CT image, we find that the maximum value of Fig 3(b) corresponds to the minimum value of Fig 4(a), and the results roughly coincide. Fig 4(b) shows the results of Fig 4(a) in ascending order. Fig 4(c) shows the first eight data points shown in Fig 4(b), which are more likely to reflect the trend in the slope change. The **Determine the Threshold** algorithm is shown in Table 3.

**Table 3:** Determine the threshold algorithm.

*Algorithm 3 Determine the Threshold*
1: Obtain Fig 4(c) by calculating the distance and fitting 2: ***while*** distance> = 0 && distance< = 20 ***do*** 3: ***if*** the point at which the slope is changed cannot be detected 4: ***then*** set the clustering threshold ***E*** = 20 5: ***else*** detect the maximum point of the change in slope ***max*** 6: ***then*** obtain the value of the distance (***dist***) corresponding to the point (***max***) and set ***E*** = ***dist*** 7: ***end if*** 8: ***end while***

After executing the **Determine the Threshold** algorithm, the clustering thresholds for the three types of lung nodules were 5.48, 6.73, and 12.65. In general, the clustering threshold for cavitary nodules will be slightly larger. In contrast, the clustering threshold for juxta-vascular nodules will be smaller and exhibit a great relationship with the difference in the CT image gray level and the nodule location.

In addition, the accuracy of the clustering threshold further ensures that our method can accurately segment cavitary nodules and juxta-vascular nodules, as mainly reflected by two aspects. First, the cavities are effectively segmented as a part of the nodule, which ensures the integrity of cavitary nodule segmentation. Second, the effective separation of blood vessels and nodules ensures the accuracy of juxta-vascular nodule segmentation.

**3. Improved DBSCAN algorithm for superpixel sequence image clustering:** Compared with the traditional DBSCAN clustering algorithm, the advantages of the Improved DBSCAN algorithm are mainly reflected by three aspects. First, the clustering algorithm is based on superpixels rather than pixels. Second, by constructing the adaptive weight coefficient in the calculation of the distance between superpixels, initiation of the clustering block can be obtained. Thus, the purpose of clustering can be achieved only for lung nodules. Third, by fitting the distance between superpixels and detecting the change in slope, the clustering threshold can be obtained, achieving the effect of the adaptive threshold and improving the accuracy of segmentation.

The **Improved DBSCAN** algorithm is shown in Table 4.

**Table 4:** Improved DBSCAN algorithm.

*Algorithm 4 Improved DBSCAN*
1: Obtain starting clustering block sequences (***indexes***) by calculating the sum of the distance between the respective superpixel blocks. 2: Obtain clustering threshold sequences (***Ts***) by performing the ***Determine the Threshold*** algorithm. 3: ***Repeat*** 4: Extract the ***index*** from the ***indexes*** and obtain the corresponding threshold ***T*** from ***Ts***. 5: Initialize the visit array (***visited***) for all ***0***. 6: ***if index*** is not visited 7: ***then visited(index) = 1*** is set to be visited. In the adjacency matrix of the ***index***, the label array (***neighbors***) of the adjacent blocks of the similarity distance ***d(b***_***index***_, ***b***_***j***_***)*** (***j*** is the label number of the adjacent superpixels with ***index***) is searched, which is less than the threshold ***T***. 8: ***Repeat*** 9: Remove a label number in the array ***neighbors*** denoted as ***i***. 10: ***if i*** is not visited 11: ***then visited(i) = 1*** is set to be visited. As in step six, in the adjacency matrix of ***i***, searching the label array (***n***) of the adjacent blocks of the similarity distance ***d(b***_***i***_, ***b***_***j***_***)*** (***j*** is the label number of the adjacent superpixels with ***i***) is less than the threshold ***T*** and then merges ***n*** into ***neighbors***. 12: ***end if*** 13: ***Until*** all data for the ***neighbors*** are executed 14: Merge superpixels marked as already visited and obtain the lung nodule masks. 15: ***end if*** 16: ***Until*** all values for the ***indexes*** are performed 17: Obtain the lung nodule mask sequences.

#### Method for segmenting lung nodule image sequences

The overall description of the method for lung nodule image sequence segmentation is shown in Table 5.

**Table 5:** Lung nodule image sequence segmentation algorithm.

*Algorithm 5 Lung nodule image sequences segmentation algorithm*
1: Input the original CT image sequences, and initialize the number of slices of AIP reconstruction area ***S***_***r***_ and the total number of AIP reconstruction sequence images ***N***_***r***_. 2: Lung parenchyma image sequence segmentation. 3: Obtain the AIP sequence images ***AIP***_***i***_, i ∈ [1,***N***_***r***_]. The lung parenchyma image sequences are labeled ${\left\{CT_{ij}\right\}}_{j=1}^{S_{r}}$, with i ∈ [1,***N***_***r***_], and j ∈ [1,***S***_***r***_]; j represents the corresponding relationships of the reconstructed sequence number. 4: For lung parenchyma images labeled {*CT*_*i*1_}, with i ∈ [1,***N***_***r***_], execute the following: 1) Compared with the ***AIP***_***i***_ image sequence after multi-scale dot enhancement, retain the dot-like object. 2) Superpixel sequence image segmentation (***HMSLIC***). 3) Calculate the distance between superpixels and perform the ***Determine the Threshold*** algorithm to obtain the starting clustering point, the starting clustering block and the clustering threshold, denoted as [*x*_*i*_, *y*_*i*_], {*S*_*i*1_} and {*E*_*i*1_}. 5: For lung parenchyma images labeled ${\left\{CT_{ij}\right\}}_{j=2}^{S_{r}}$, with i ∈ [1,***N***_***r***_], and j ∈ [2,***S***_***r***_], take [*x*_*i*_, *y*_*i*_] as the center and 30 mm as the side to extract ROIs as follows: 1) Convert coordinates [*x*_*i*_, *y*_*i*_] of the clustering starting point to the ROI as [*X*_*i*_, *Y*_*i*_]. 2) Superpixel sequence image segmentation (***HMSLIC***). 3) Mark the label of the pixel point [*X*_*i*_, *Y*_*i*_], which belongs to the superpixel as the starting clustering block ${\left\{S_{ij}\right\}}_{j=2}^{S_{r}}$. 4) Obtain the clustering threshold by performing the ***Determine the Threshold*** algorithm labeled as {Eij}j=2Sr. 6: According to the clustering starting blocks ${\left\{S_{ij}\right\}}_{j=1}^{S_{r}}$ and clustering thresholds ${\left\{E_{ij}\right\}}_{j=1}^{S_{r}}$, perform the ***Improved DBSCAN*** algorithm to obtain the lung nodule mask sequence ${\left\{M_{ij}\right\}}_{j=1}^{S_{r}}$. 7: According to ${\left\{M_{ij}\right\}}_{j=1}^{S_{r}}$, sequential segmentation of all lung nodule images.

## Results and discussion

To evaluate the overall performance of our segmentation method for the three types of nodules, we conducted many contrast experiments using our method, region growing(RG) [26], pcnn-pulse coupled neural network (PCNN) [27], KM [28], FCM [29], particle swarm optimization (PSO)-self-generating neural network (SGNN) [15] and flowing entropy and geodesic distance (FEGD) [7]. All the experimental results were compared with the manual segmentation results. All the algorithms in the experiment are presented by Visual Studio 2013, MATLAB R2014b, ITK 4.7.2, or VTK6.3.0 and were performed on a PC machine with a 3.4 GHz Intel (R) Core (M) i7-4770 processor with 8 GB of RAM.

### Qualitative evaluation

In the present analysis, for the lung CT image sequences of solitary pulmonary nodules, cavitary nodules and juxta-vascular nodules, we compared the segmentation results of our method, RG, PCNN, KM, FCM, PSO-SGNN and FEGD. The parameter settings of our method are shown in Table 6.

**Table 6:** The parameter settings of our method.

Index	Parameters	Values
1	*S*_*r*_, *t* ***in AIP***	4, 0.6 or 1 or 1.3 or 1.5
2	*σ*, *β*, *N*, *d*_*min*_, *d*_*max*_ ***in Hessian***	0.5, 15, 8, 3, 30
3	*K*, *m*, ***r***, ***E in HMSLIC***	1800 or 30~60 (ROIs), 25, 1, 0.0001
4	*w* ***in Distance Calculation***	adaptive
5	***MinPts***, ***index***, ***T in Improved DBSCAN***	1, adaptive, adaptive

By analyzing all the experimental data, we discovered that the average number of CT image sequences containing nodules is twelve. Moreover, considering that the changes in the relative position of nodules between the four CT images were very small, we set parameter *S*_*r*_ = 4, which has a better effect on sequence segmentation.

**Fig 5:**
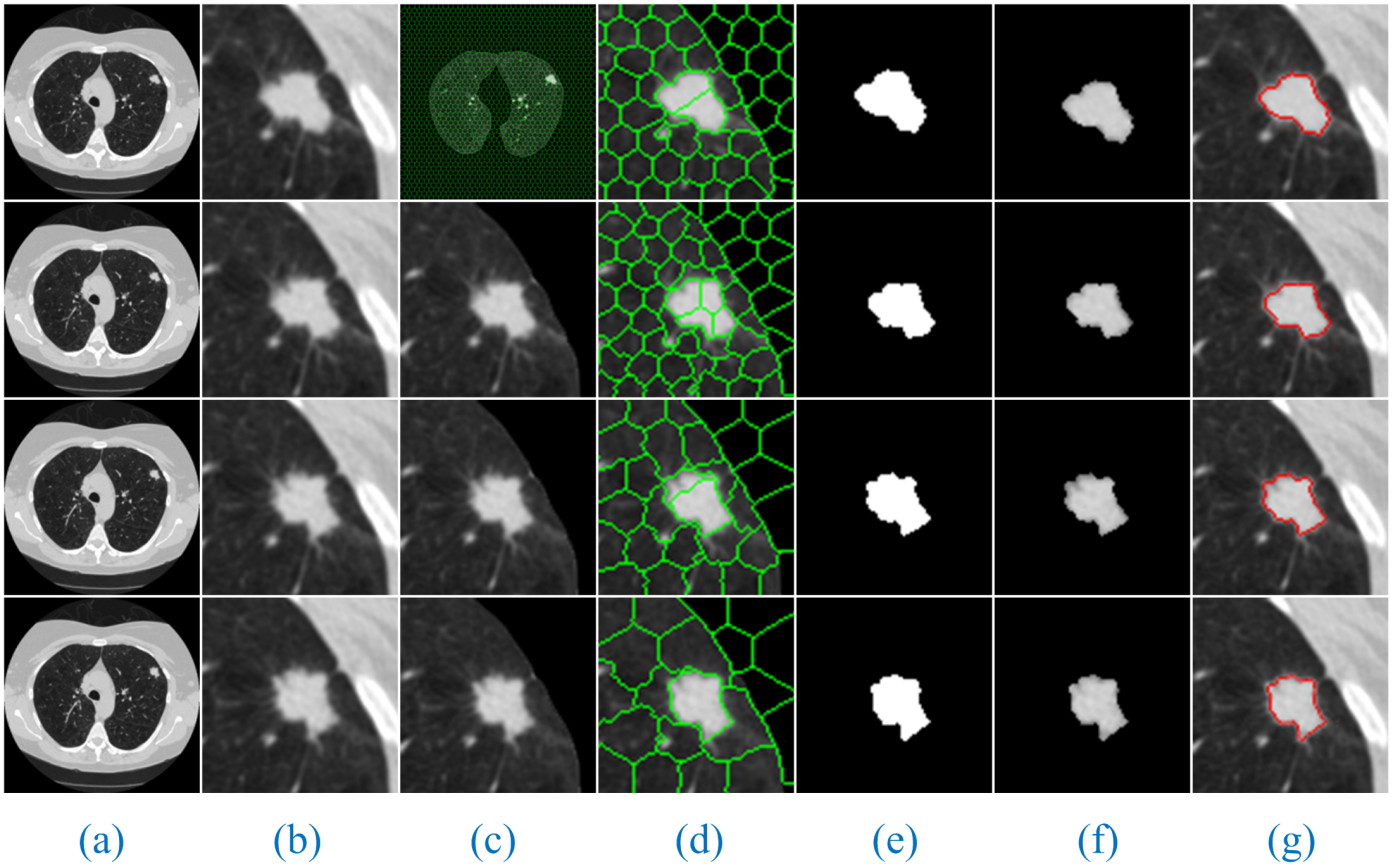
The sequence segmentation results of our method for solitary pulmonary nodules. Column (a) shows the original lung CT sequence images, (b) shows the results of the local enlargement of (a), (c) shows the segmentation results of HMSLIC (first image) and the extraction of ROI images (other images), (d) shows the local enlargement of (c) (first image) and the segmentation results of the ROI images using HMSLIC (other images), (e) shows lung nodule image mask sequences obtained by the Improved DBSCAN algorithm, (f) and (g) present the final results using our method and manual segmentation by experts.

**Fig 6:**
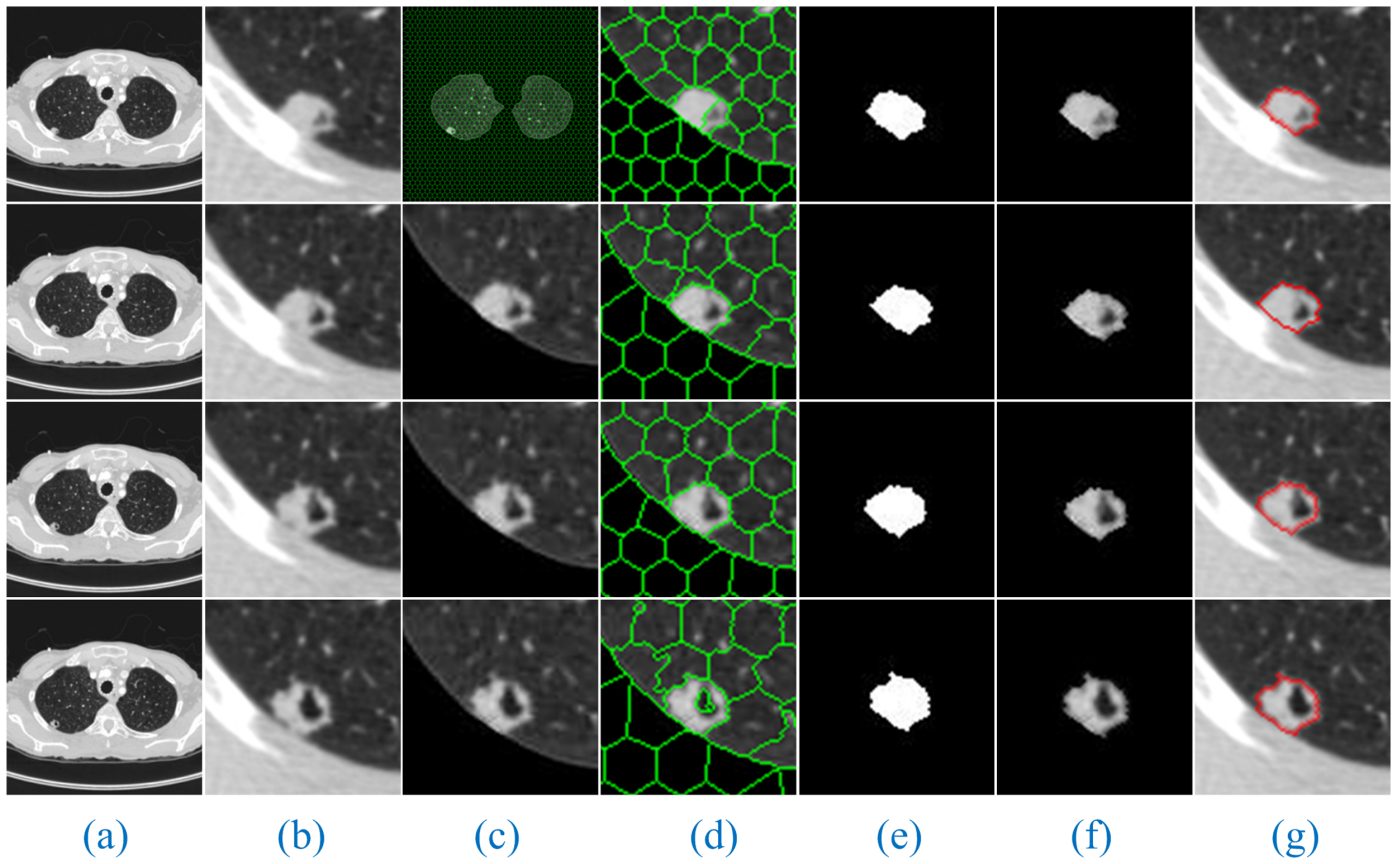
The sequence segmentation results of our method for cavitary nodules. The detailed descriptions are the same as in Fig 5.

**Fig 7:**
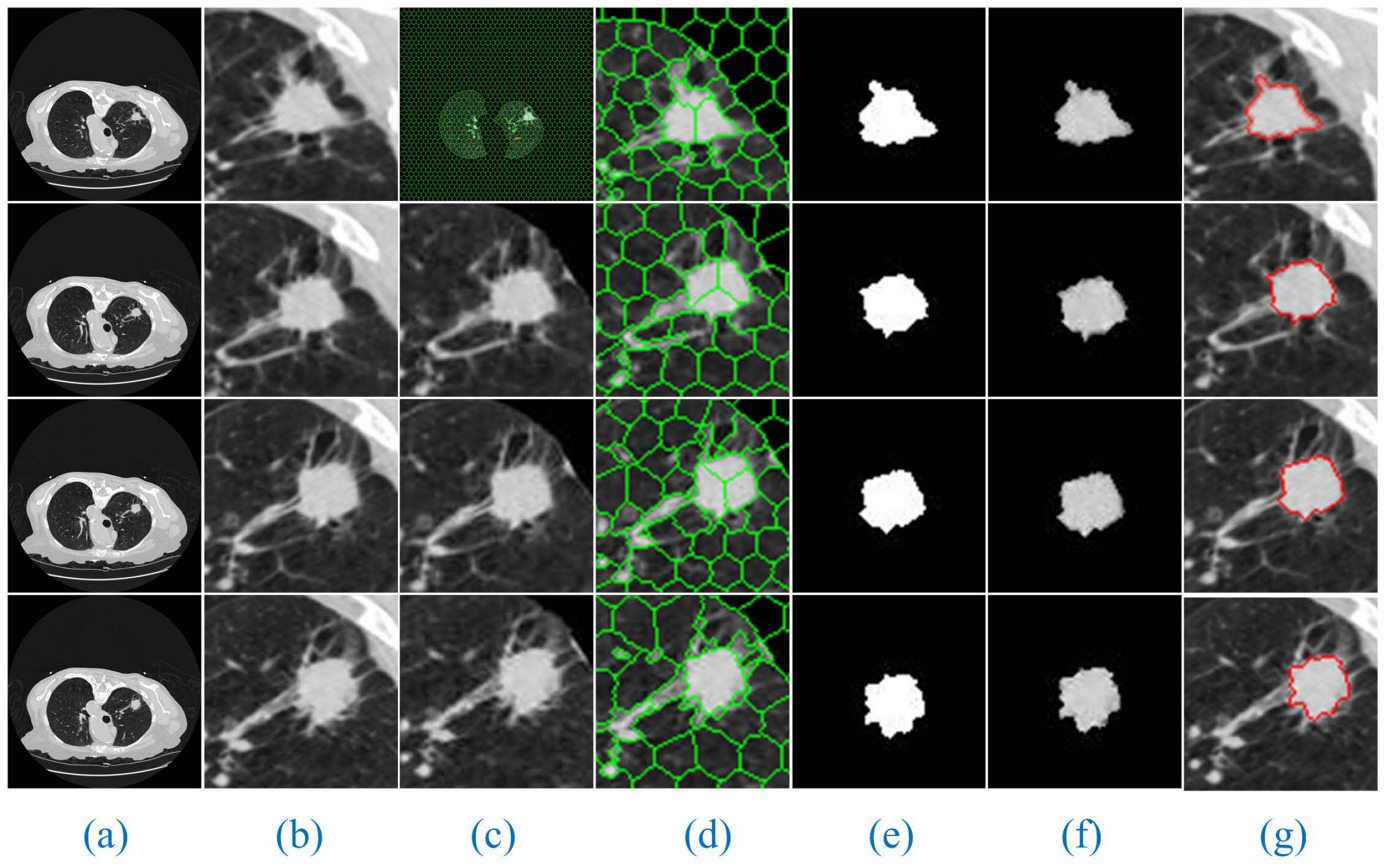
The sequence segmentation results of our method for juxta-vascular nodules. The detailed descriptions are the same as in Fig 5.

Additionally, since the number of lung CT sequence images was large, this paper only selected three AIP reconstruction sections of three types of nodule images to demonstrate and illustrate the sequence segmentation process, as shown in Figs 5–7. The other AIP reconstruction sections were similar.

As shown in Figs 5–7, a full account of the correlation between the front and back CT images of each AIP reconstruction section is provided. The coordinates of the clustering starting points of the front and back images are transmitted well. Therefore, in the single AIP reconstruction section, we extract only ROI sequences for localization and direct operation, except for the first image. This process can significantly accelerate the speed of the algorithm and eliminate the interference of most of the background noise.

**Fig 8:**
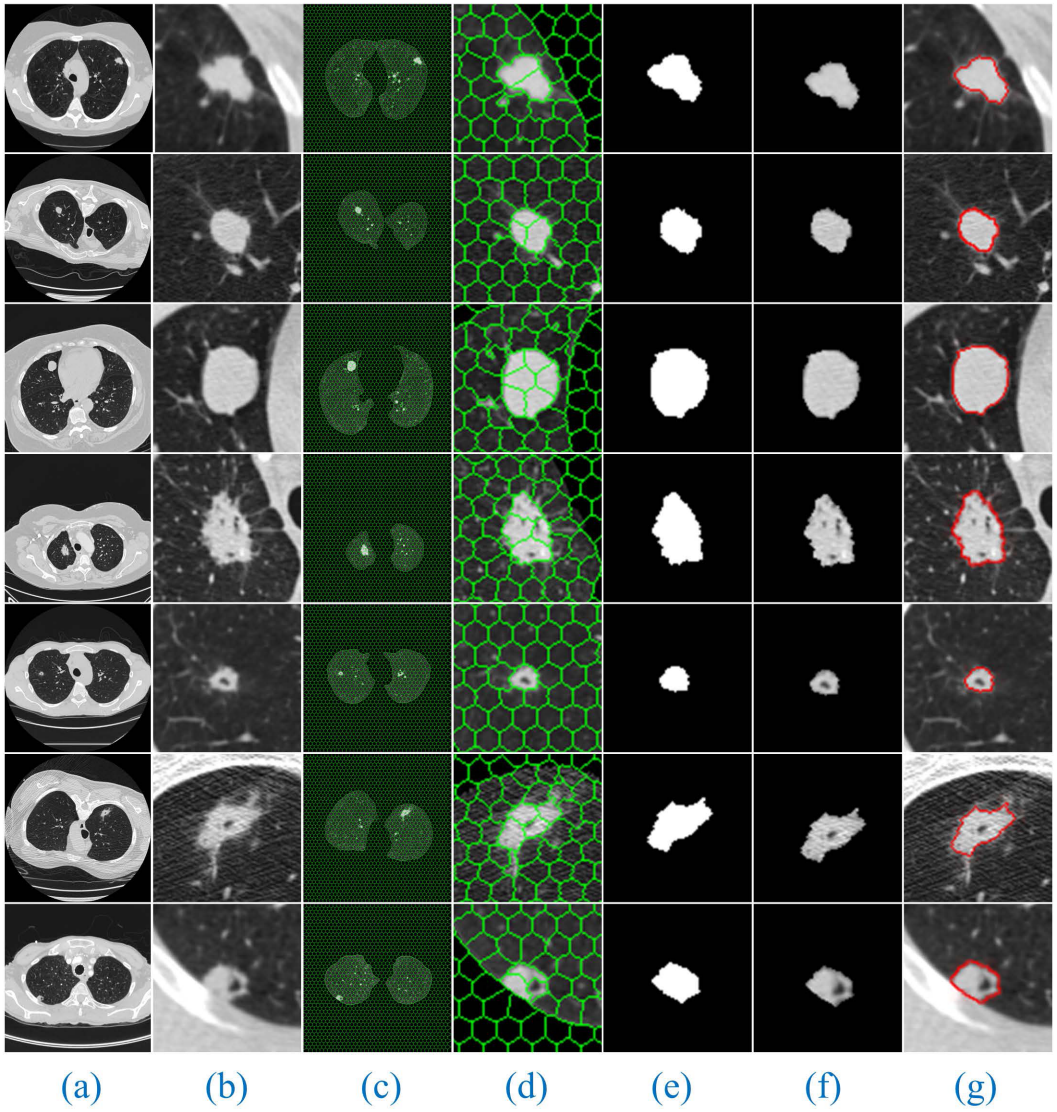
The segmentation results of our method for solitary pulmonary nodules and cavitary nodules. Column (a) shows the original lung CT images, (b) shows the results of the local enlargement of (a), (c) shows the segmentation results of HMSLIC, (d) shows the results of the local enlargement of (c), (e) shows the lung nodule image masks obtained by the Improved DBSCAN algorithm, and (f) and (g) present the final results using our method and manual segmentation by experts.

**Fig 9:**
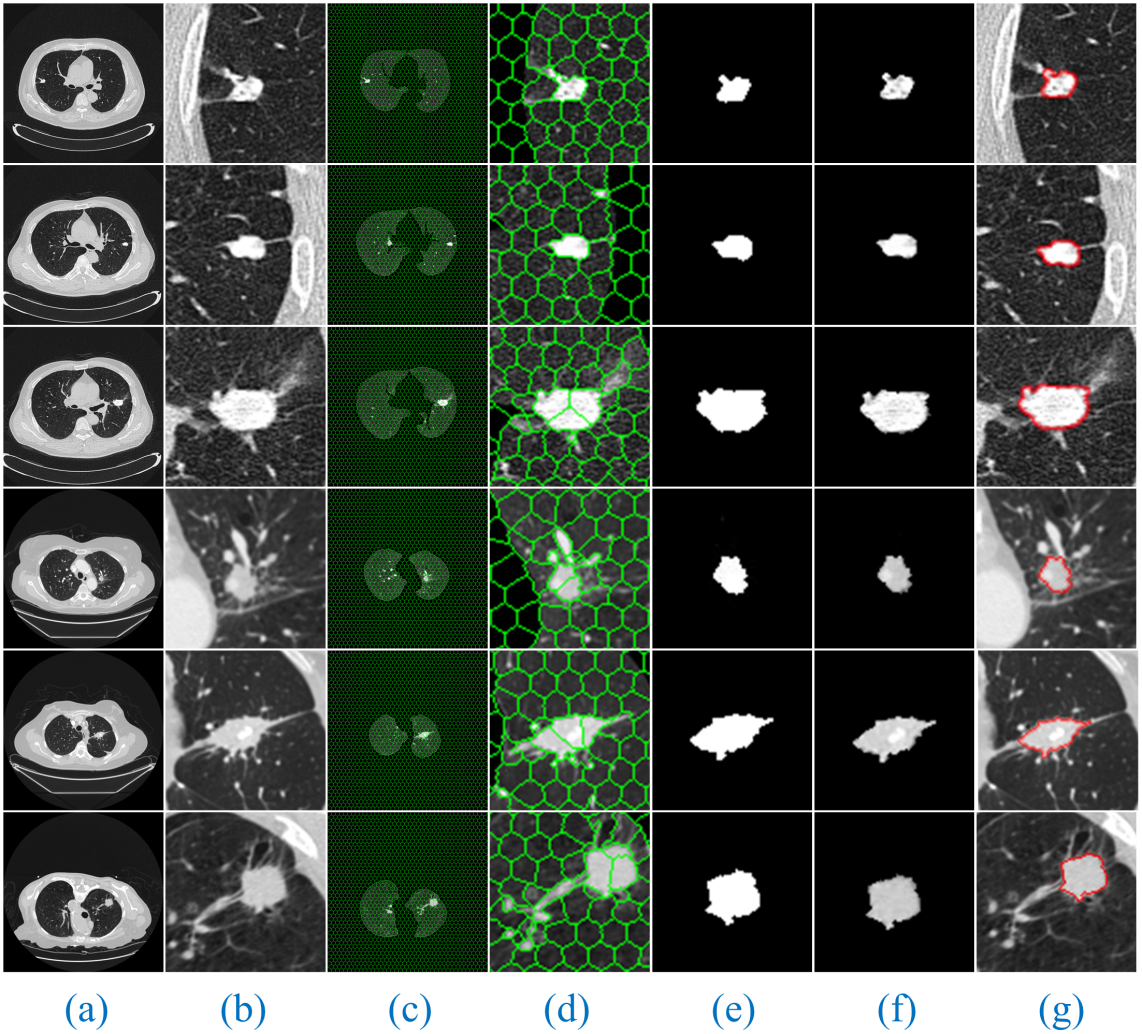
The segmentation results of our method for juxta-vascular nodules. The detailed descriptions are the same as shown in Fig 8.

Furthermore, Fig 8 shows the segmentation results of our method for solitary pulmonary nodules and cavitary nodules. Fig 9 shows the segmentation results of our method for juxta-vascular nodules.

**Fig 10:**
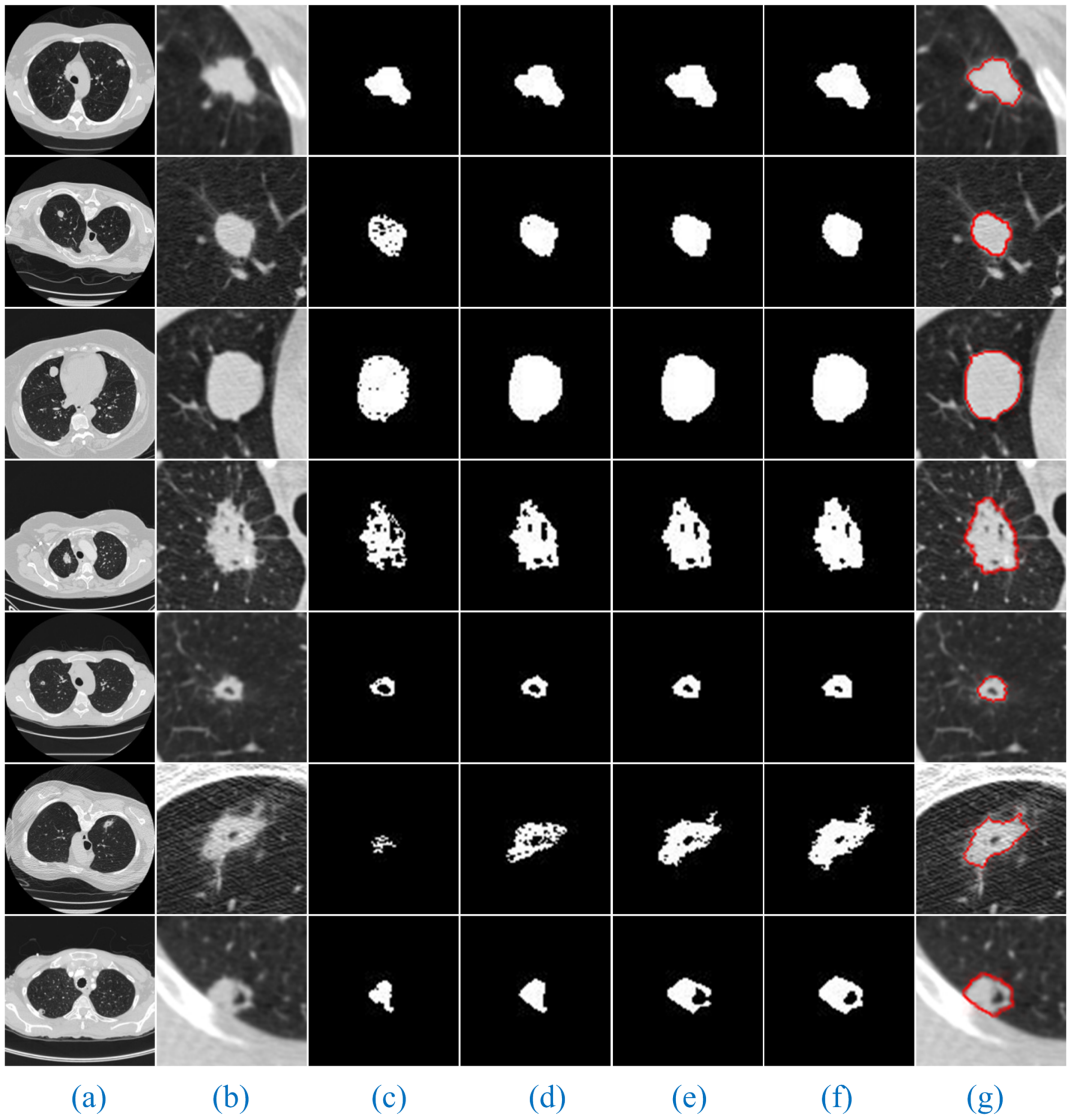
The segmentation results of RG for solitary pulmonary nodules and cavitary nodules. Column (a) shows the original lung CT images, (b) shows the results of the local enlargement of (a), (c)-(f) show the segmentation results of lung nodule image masks when the gray threshold is 0.05, 0.1, 0.15 and 0.2 (/255), and (g) shows the results of manual segmentation by experts.

**Fig 11:**
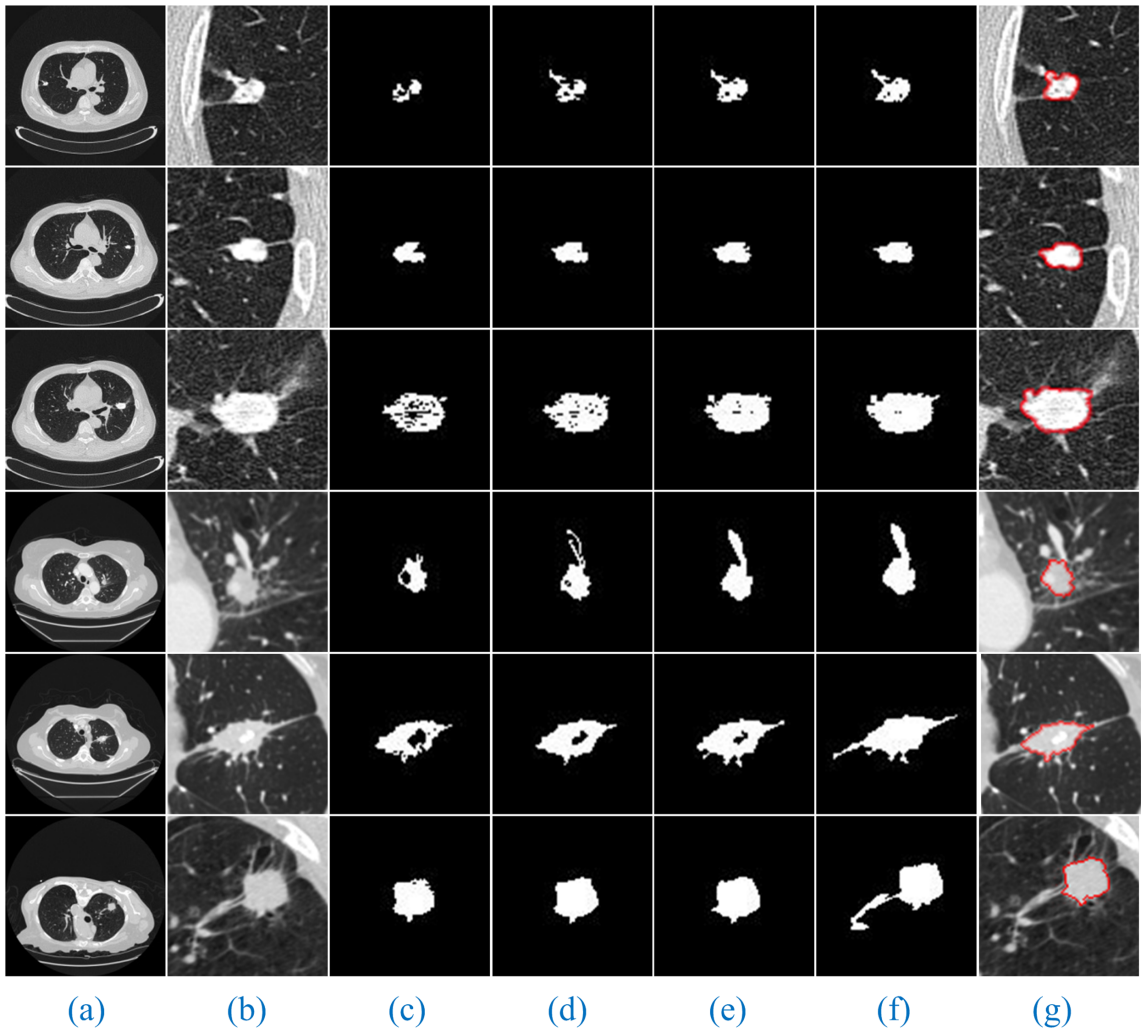
The segmentation results of RG for juxta-vascular nodules. The detailed descriptions are the same as presented in Fig 10.

When using RG, we must select the ***seed points***. In the experiment, the coordinates of the seed points corresponding to the lung CT images in (Figs 8 and 9, Column (a)) are (394, 193), (188, 193), (166, 212), (211, 335), (131, 237), (359, 197), (120, 321), (126, 248), (391, 252), (368, 241), (314, 286), (304, 267) and (340, 287) from the top to the bottom. In addition, then those points were used as the starting points of RG to segment the lung nodule images. Moreover, we discovered that different gray thresholds have different segmentation effects. (Figs 10 and 11, Column (c)-(f)) correspond to the segmentation results of the ***gray threshold*** of 0.05, 0.1, 0.15 and 0.2 (/255), respectively. We noticed that the gray threshold of the best segmentation effect for solitary pulmonary nodules and cavitary nodules was 0.2, and that for juxta-vascular nodules was 0.15.

**Fig 12:**
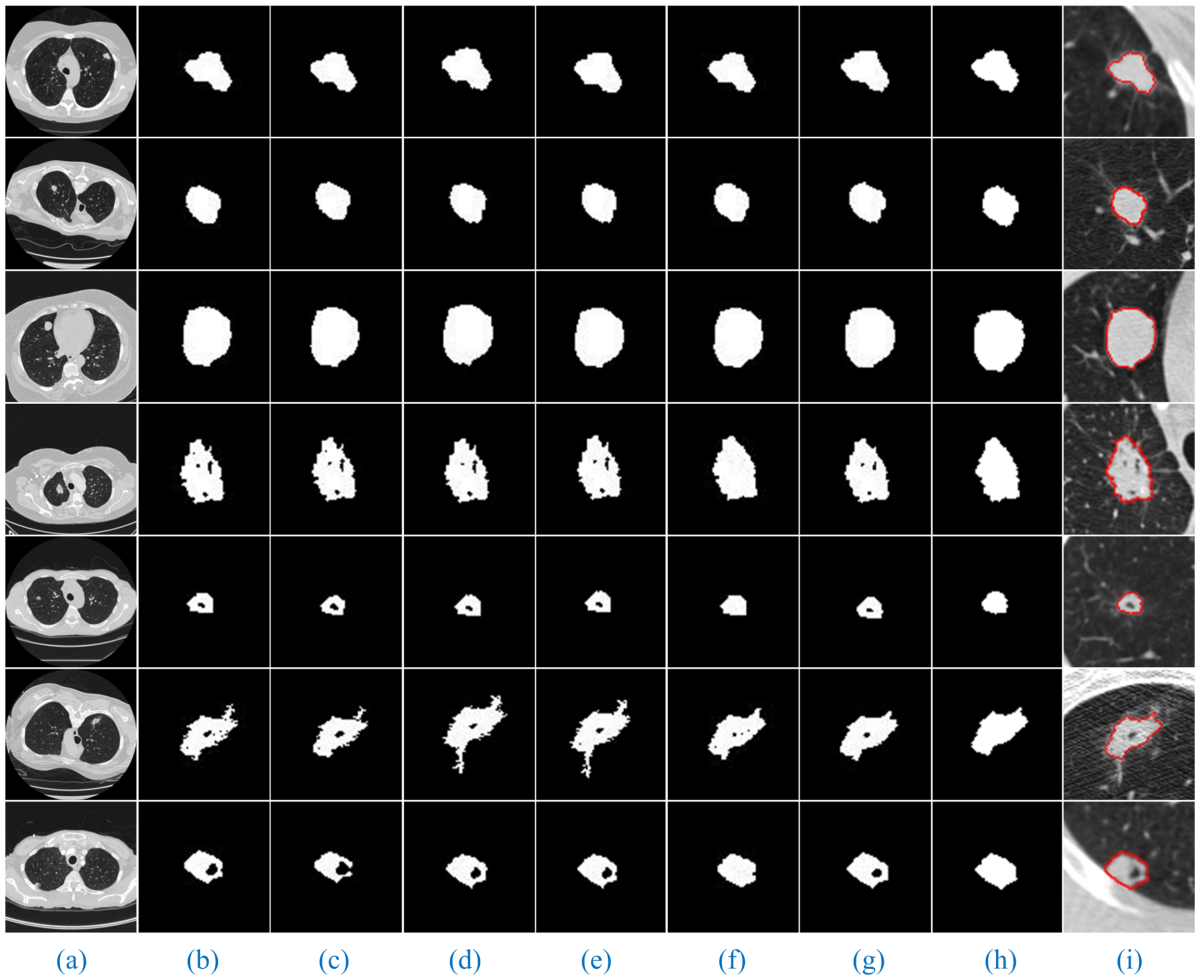
Comparison of the segmentation results of seven methods for solitary pulmonary nodules and cavitary nodules. Column (a) shows the original lung CT images, (b)-(h) show the results of the lung nodule image masks using RG (gray threshold is 0.2), PCNN, KM, FCM, PSO-SGNN, FEGD and our method, and (i) shows the results of manual segmentation by experts.

**Fig 13:**
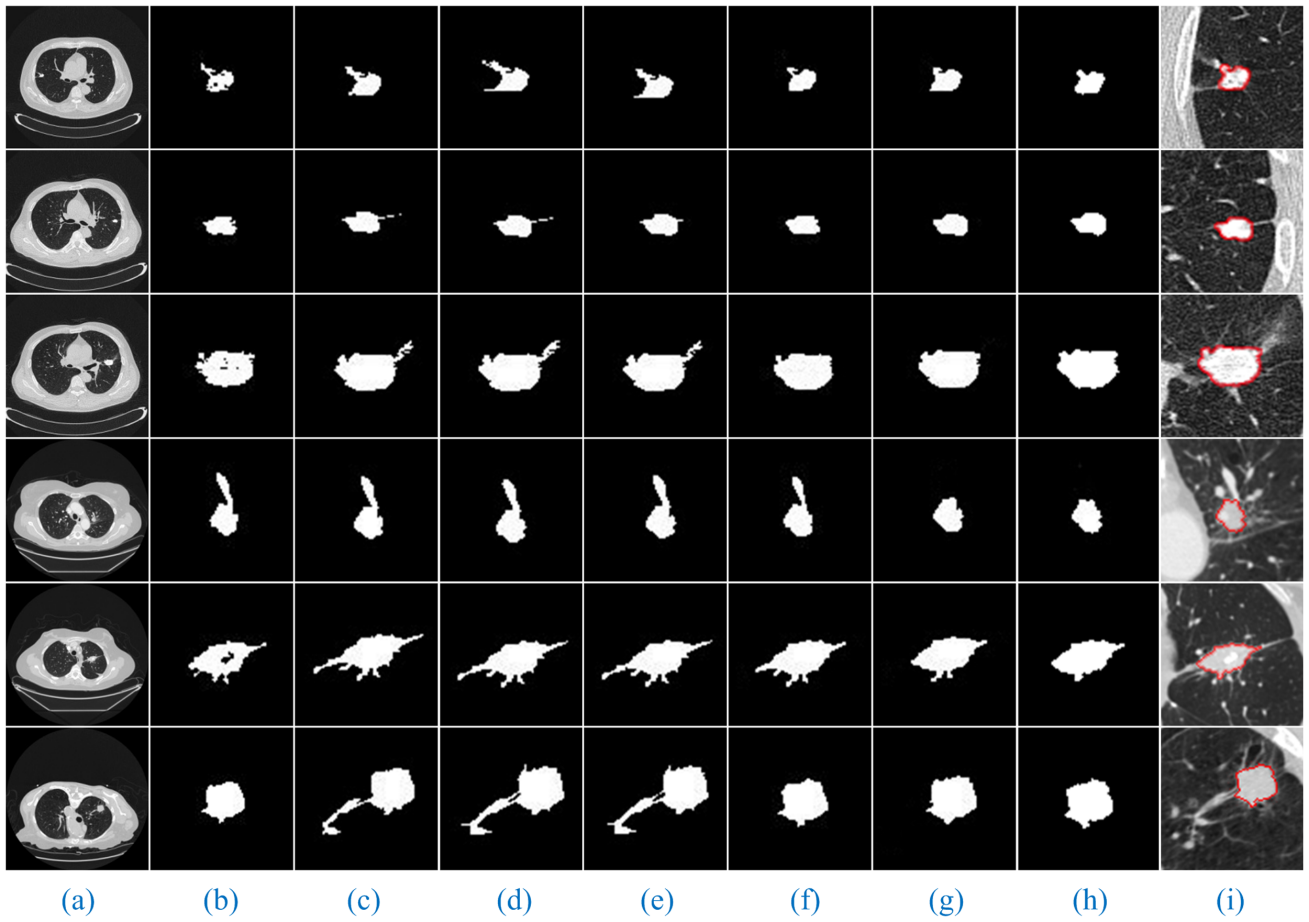
Comparison of the segmentation results of seven methods for juxta-vascular nodules. Column (b) shows the results of the lung nodule image masks using RG (gray threshold of 0.15). The other detailed descriptions are the same as in Fig 12.

For the lung CT images of solitary pulmonary nodules, cavitary nodules and juxta-vascular nodules, we compared the segmentation results of seven algorithms with the manual segmentation results (Figs 12 and 13).

When using PCNN, the parameter settings are as follows: ***linking strength coefficient*** = 0.32, ***threshold amplitude coefficient*** = 200, ***decay term for threshold*** = 0.35, and ***weight matrix*** = [0.5 1 0.5; 1 0 1; 0.5 1 0.5]. At this point, better segmentation results were obtained, as shown in (Figs 12 and 13, Column (c)).

When using KM to segment lung CT images, we set the ***convergence threshold*** to 0.00001; that is, if the difference in the mean of the gray of the two times is less than the value, then the algorithm ends. The best segmentation effects obtained at this point are presented in (Figs 12 and 13, Column (d)).

**Fig 14:**
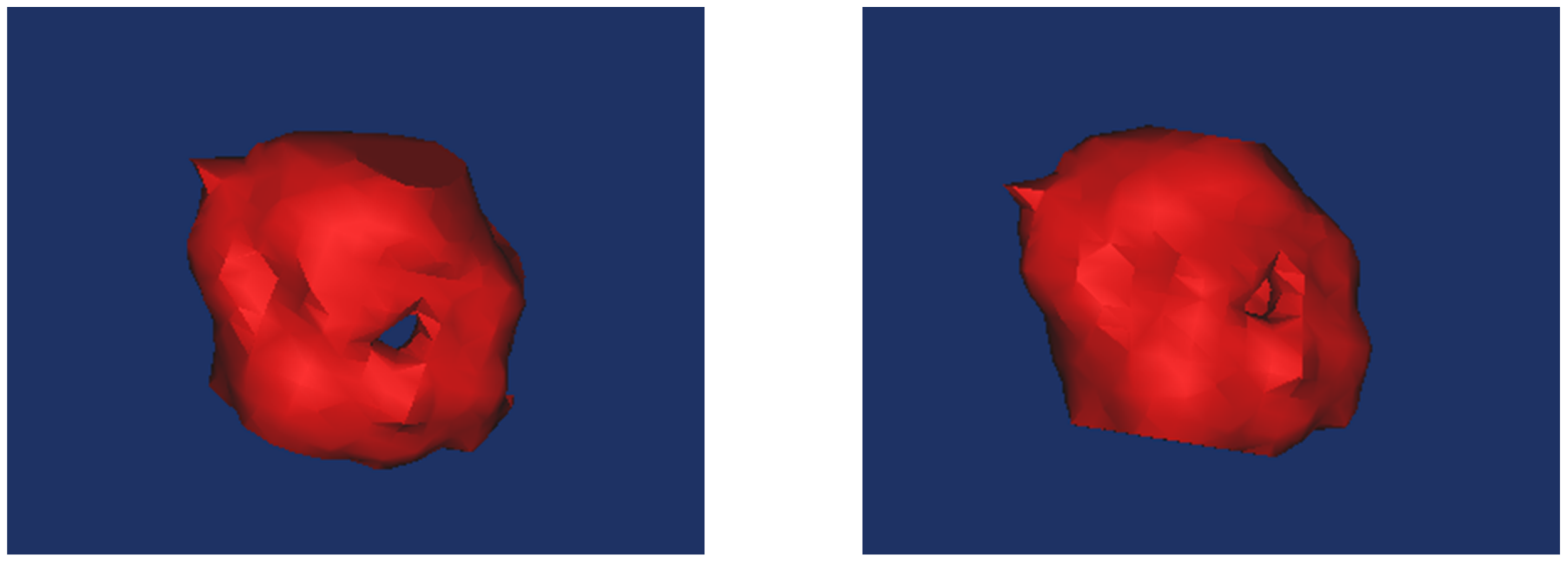
3D reconstruction of a cavitary nodule.

**Fig 15:**
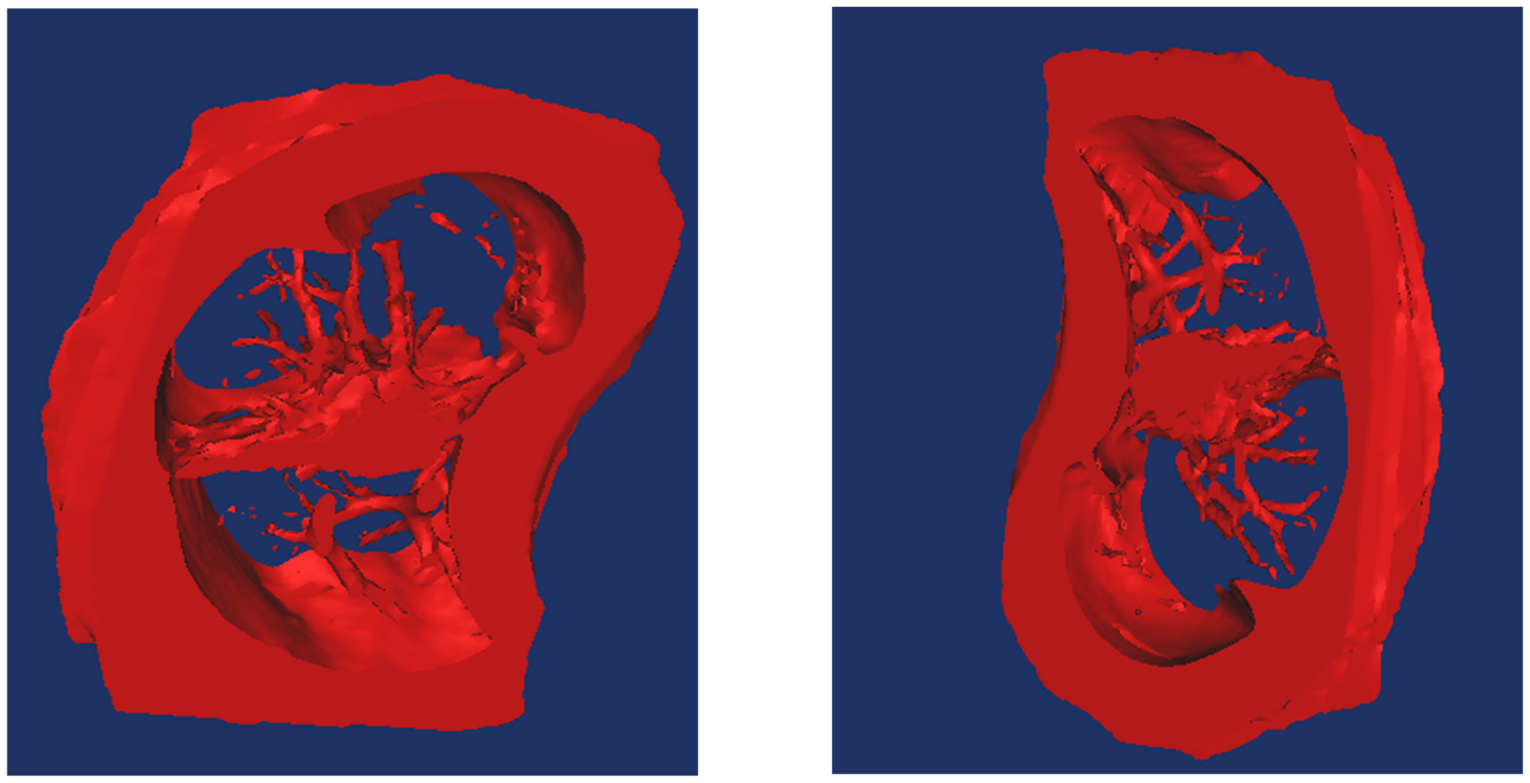
3D reconstruction of juxta-vascular nodules and surrounding non-target tissues.

When using FCM to segment the images, we set the ***change rate threshold*** of the clustering center to 0.1%; that is, if the change in the rate of the value of the clustering center is less than the value, then the algorithm stops. The best segmentation effects at this point are shown (Figs 12 and 13, Column (e)). Moreover, Fig 14 shows the 3D reconstruction of cavitary nodules. Fig 15 shows the 3D reconstruction of juxta-vascular nodules and surrounding non-target tissues.

When using PSO-SGNN to segment CT images, we assume that the number of SGNN samples is n. Thus, the PSO parameter settings are as follows: ***particle position*** = [1, n], ***particle speed*** = [1, n/5], ***maximum iterations*** = 2n, ***inertia weight*** = 0.9~0.4, and ***particle weight coefficient*** = 2. Additionally, the SGNF is generated using the ***gray scale*** and ***the coordinates*** as the two properties of the sample. At this point, better segmentation effects are obtained, as shown in (Figs 12 and 13, Column (f)).

When using FEGD to segment the images, the parameter settings are as follows: ***number of KM clustering*** = 2, ***neighborhood scale of flowing entropy*** = 2 or 3. The best segmentation results at this point are shown in (Figs 12 and 13, Column (g)).

From the segmentation results, we can observe that the advantages of our method are especially obvious for the lung images with cavitary nodules and juxta-vascular nodules.

For the lung CT image sequences with solitary pulmonary nodules, the segmentation results of our method and the other six segmentation algorithms are basically consistent with the manual segmentation results, indicating that all the segmentation results are accurate.

For the lung CT image sequences with cavitary nodules, the segmentation results of RG, PCNN, KM, FCM and FEGD will miss the nodule cavities. We can see that the segmentation performance of those algorithms is poor. Although PSO-SGNN achieved better segmentation results, the boundary information for the segmentation results is partially missing (as shown in (Fig 12, Rows (4–5))). Simultaneously, SGNN does not entirely the nodule cavities when the lung CT image is more affected by noise (as shown in (Fig 12, Row (6))) or the cavities are at the edge of the nodule (as shown in (Fig 12, Row (7))). However, the HMSLIC algorithm can better preserve the boundary information, and the Improved DBSCAN clustering algorithm is insensitive to noise in our method. Thereby, the segmentation results of our method will contain the entire cavities and will be more consistent with the manual segmentation results by experts, ensuring the integrity of the segmentation of cavitary nodules.

For the lung CT image sequences with juxta-vascular nodules, the segmentation results of RG, PCNN, KM and FCM are inaccurate and will include blood vessels on the nodules. Additionally, the segmentation results of RG (gray threshold of 0.15) and PSO-SGNN are better than those of PCNN, KM and FCM but still include some blood vessels on the nodules. Additionally, the segmentation effect of FEGD is better with the retention of a small amount of edge leakage (as shown in (Fig 13, Row (1–4 and 6))), and the segmentation results of FEGD will partially include blood vessels in certain lung CT images (as shown in (Fig 13, Row (5))) because FEGD cannot account for the circular area of the vascular cross-section. However, our method can effectively separate blood vessels and nodules while preserving more boundary information, indicating that our method has the best segmentation effect for this type of nodule.

The experimental results show that by using our method, despite the appearance of non-target tissues, such as cavities and blood vessels, the nodules will be correctly segmented from the lung CT image sequences. Additionally, compared with manual segmentation, our method is accurate and the resulting edge is smooth. This result further indicates that our method can more stably, completely and accurately segment those three types of lung nodule image sequences than the other tested methods and has the best segmentation effects.

### Quantitative comparisons

Quantitative comparisons were conducted to further verify the validity and generality of our method. In the experiment, we evaluated seven segmentation algorithms using the probabilistic rand index (PRI), global consistency error (GCE), Variation of Information (VoI) and time complexity. Moreover, we hypothesized that the original CT image ***S*** encompasses *K* pixels and that the reference ***S***_***a***_ and the actual segmentation results ***S***_***b***_ encompass *M* segmentation blocks $\left\{S_{1}^{a},\;S_{2}^{a},\;\ldots ,\;S_{M}^{a}\right\}$ and *N* segmentation blocks $\left\{S_{1}^{b},\;S_{2}^{b},\;\ldots ,\;S_{N}^{b}\right\}$, respectively.

The PRI is a parameter for evaluating the consistency of attributes of symbiosis between the actual segmentation results and the reference [30]. The PRI is defined as

$$PRI(S_{b},\{S_{M}^{a}\})=1-\left[(\sum_{i}K_{i.}^{2}+\sum_{j}K_{.j}^{2})/2-\sum_{i,j}K_{ij}^{2}\right]/K(K-1)/2]$$ (15)

where *K*_*ij*_ represents the number of pixels marked as i in ***S***_***a***_ and marked as j in ***S***_***b***_. *K*_*i*._ represents the number of pixels marked as i in ***S***_***a***_. *K*_.*j*_ represents the number of pixels marked as j in ***S***_***b***_.

**Fig 16:**
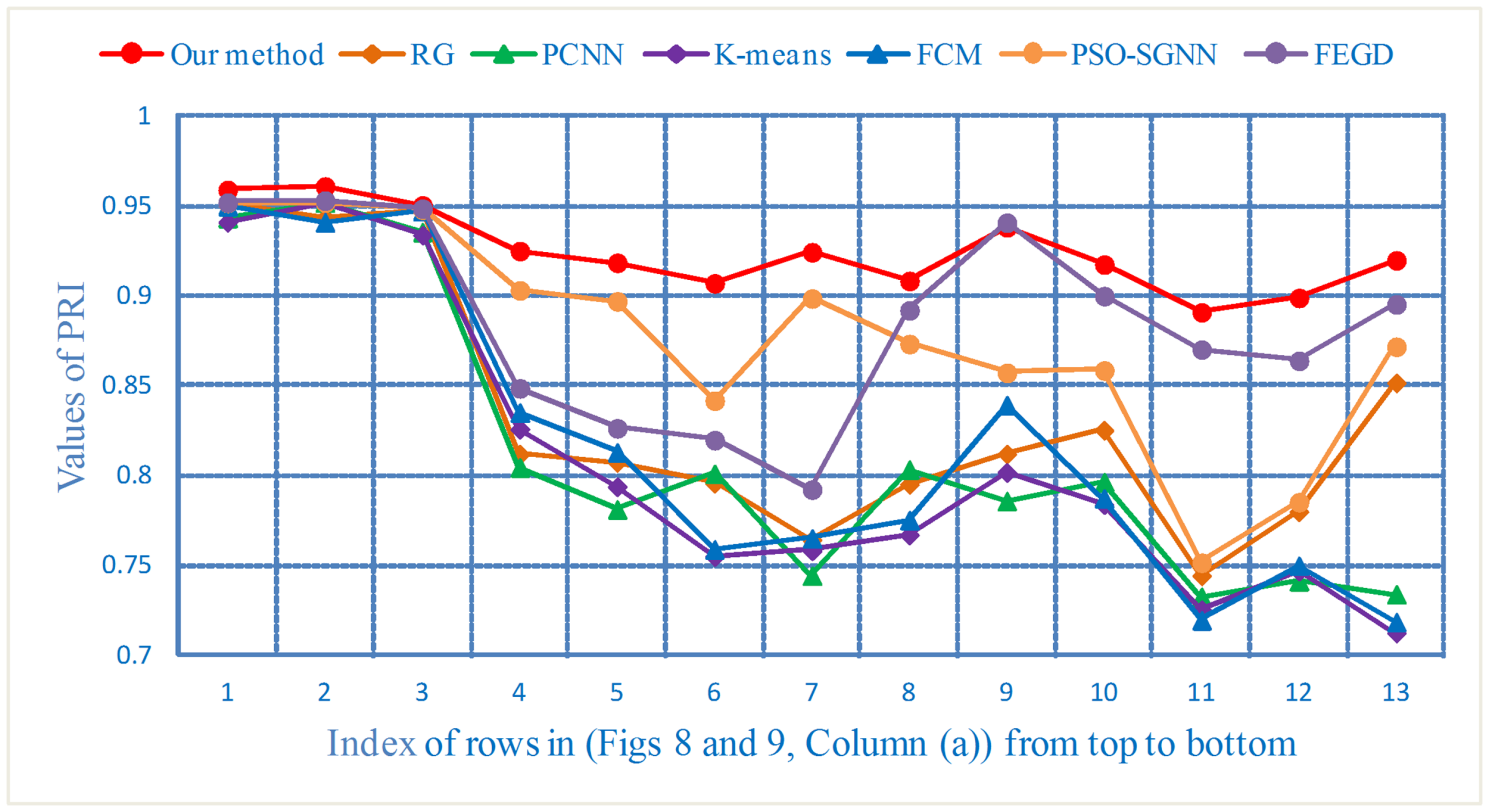
The PRI curves of the segmentation results of three types of nodule images.

The PRI values lie in the range of [0, 1], and the larger the value, the closer the segmentation results will be to the manual segmentation results derived by experts. For (Figs 8 and 9, Column (a)), the PRI curves are shown in Fig 16.

The GCE is defined based on the local refinement error [31], which is used to measure the degree that a segmentation result can be considered a subset of the other.

For pixel *P*_*i*_ in the original CT image, the reference and the actual segmentation results satisfy $P_{i}\in S_{m}^{a}$ and $P_{i}\in S_{n}^{b}$, respectively. If $S_{n}^{b}\in S_{m}^{a}$, then the local refinement error is defined as

$$E(S_{m}^{a},S_{n}^{b},P_{i})=<G(S_{m}^{a},P_{i})-G(S_{n}^{b},P_{i})>/<G(S_{m}^{a},P_{i})>$$ (16)

where < G > represents the number of elements (corresponding pixels in this paper) in the set G, and “-” represents the subtraction operation. Moreover, the local refinement error also satisfies Eqs (17) and (18):

$$E(S_{m}^{a},S_{n}^{b},P_{i})\neq E(S_{n}^{b},S_{m}^{a},P_{i})$$ (17)

$$E(S_{m}^{a},S_{n}^{b},P_{i})=\{\begin{array}{cc} 0 & S_{m}^{a}\in S_{n}^{b}\\ \neq 0 & other \end{array}$$ (18)

According to the local refinement error of every pixel in each direction in the original CT image, the GCE is defined as

$$GCE(S_{a},S_{b})=\frac{1}{K}\text{min}\left\{\sum_{i}E(S_{a},S_{b},P_{i}),\sum_{i}E(S_{b},S_{a},P_{i})\right\}$$ (19)

**Fig 17:**
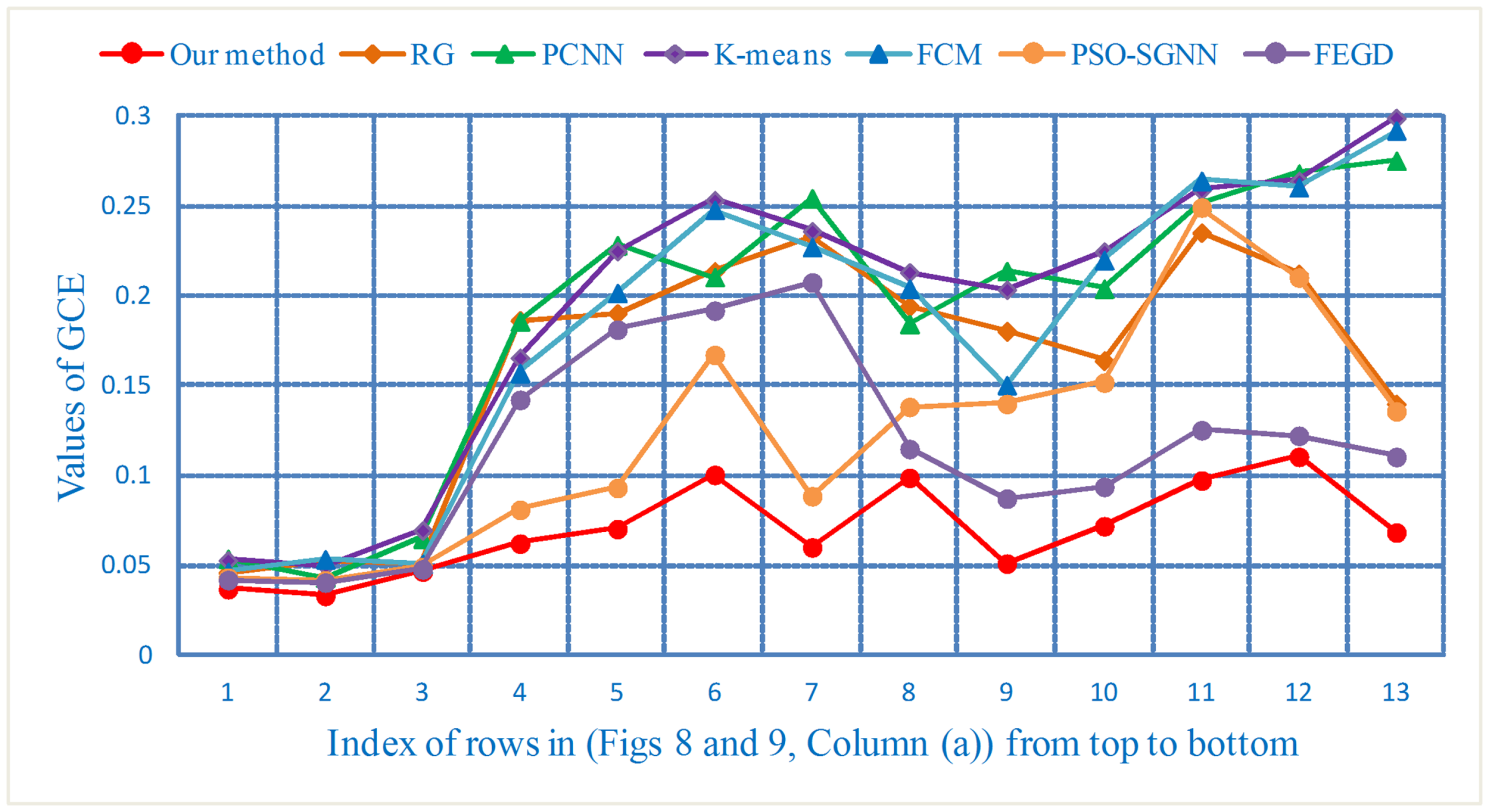
The GCE curves of the segmentation results for three type of nodule images.

The GCE values lie in the range of [0, 1], and the smaller the value, the better will be the segmentation results. For (Figs 8 and 9, Column (a)), the GCE curves are shown in Fig 17.

VoI is a criterion for measuring the amount of information lost and gained during the change from clustering to clustering [32].

All the segmentation blocks in the reference ***S***_***a***_ satisfy Eq (20):

$$S_{i}^{a}\cap S_{j}^{a}=\Phi ,\;i=1,\;2,\ldots ,M,j\neq i$$ (20)

If we assume that the total number of pixels of the m-th segmentation block $S_{m}^{a}$ in ***S***_***a***_ is *K*_*m*_, then the probability *P*(*m*) of any pixels divided into the m-th block $S_{m}^{a}$ will be denoted as:

$$P(m)=K_{m}/K$$ (21)

Moreover, the entropy [33] of ***S***_***a***_ can be obtained:

$$H(S_{a})=-\sum_{m=1}^{M}P(m)\text{log }P(m)$$ (22)

Similarly

$$P(n)=K_{n}/K$$ (23)

$$H(S_{b})=-\sum_{n=1}^{N}P(n)\text{log }P(n)$$ (24)

The joint entropy of ***S***_***a***_ and ***S***_***b***_ is

$$I(S_{a},S_{b})=\sum_{m=1}^{M}\sum_{n=1}^{N}P(m,n)\text{log }\frac{P(m,n)}{P(m)\times P(n)}$$ (25)

By using the entropy of ***S***_***a***_ and ***S***_***b***_, and the joint entropy of ***S***_***a***_ and ***S***_***b***_, the VoI is defined as

$$VoI(S_{a},S_{b})=H(S_{a})+H(S_{b})-2I(S_{a},S_{b})$$ (26)

**Fig 18:**
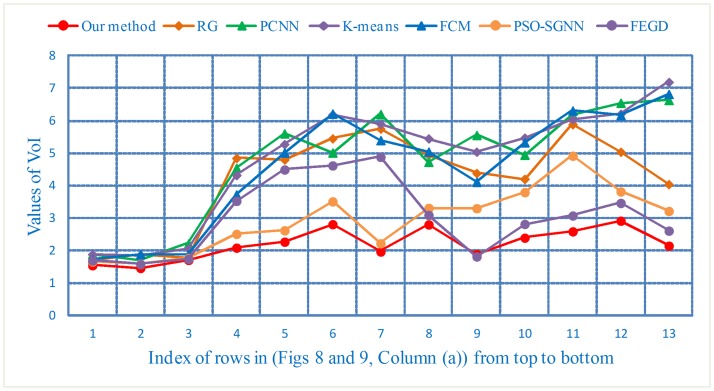
The VoI curves of the segmentation results of three types of nodule images.

The VoI values lie in the range of [0,∞), and the smaller the value, the more the actual segmentation results will be closer to the reference and the better will be the segmentation results. For (Figs 8 and 9, Column (a)), the VoI curves are shown in Fig 18.

To demonstrate the universality of our method, we further calculate the mean PRI, GCE and VoI for the segmentation results of all experimental datasets, as shown in Table 7.

**Table 7:** The mean PRI, GCE and VoI for the five algorithms in all experimental datasets.

Types	Criteria	Our method	RG	PCNN	KM	FCM	PSO-SGNN	FEGD
***Solitary- Pulmonary- Nodule***	***PRI***	0.9571	0.9477	0.9424	0.9439	0.9463	0.9509	0.9515
	***GCE***	0.0392	0.0496	0.0543	0.0587	0.0506	0.0451	0.0438
	***VoI***	1.5823	1.7943	1.9417	1.9427	1.8440	1.6847	1.6813
***Cavitary- Nodule***	***PRI***	0.9190	0.7952	0.7831	0.7836	0.7934	0.8854	0.8221
	***GCE***	0.0736	0.2062	0.2159	0.2203	0.2088	0.1078	0.1813
	***VoI***	2.2933	5.2193	5.3483	5.4273	5.0968	2.7260	4.3887
***Juxta-vascular- Nodule***	***PRI***	0.9126	0.8017	0.7659	0.7566	0.7653	0.8336	0.8941
	***GCE***	0.0833	0.1879	0.2332	0.2441	0.2321	0.1712	0.1093
	***VoI***	2.4658	4.7570	5.7705	5.9067	5.6372	3.7355	2.8226

For the lung image sequences of solitary pulmonary nodules, as shown in Table 7, the PRI, GCE and VoI show little differences among the seven segmentation methods. This result indicates that all the algorithms have good segmentation effects for this type of nodule. The GCE and VoI of the KM are greater than those of other algorithms, and the PRI of PCNN is lower than that of other algorithms. Relatively speaking, the segmentation effect of KM and PCNN is poor compared with that of the other methods.

For the lung image sequences of cavitary nodules, Table 7 illustrates that the PRI, GCE and VoI of our method reach 0.9190, 0.0736 and 2.2933, respectively. The results demonstrate that our method is significantly better than other algorithms for the segmentation of cavitary nodule sequences. Moreover, the segmentation results of PSO-SGNN are better than those of RG, PCNN, KM, FCM and FEGD.

For the lung image sequences of juxta-vascular nodules, Table 7 shows that our method also achieves the highest PRI and the lowest GCE and VoI. These findings indicate that the segmentation results of our method are optimal. Furthermore, for the three criteria presented in Table 7, the overall segmentation effect of FEGD is better than that of RG, PCNN, KM, FCM and PSO-SGNN.

From the intuitive visualization results (Figs 16–18) and objective quantification results (Table 7), we can see that our method overcomes the inaccurate segmentation of both cavitary and juxta-vascular nodules. Therefore, following the use of those operations, it is clear that the overall segmentation effect of our method is much better than that of other typical segmentation algorithms. In addition, although little image preprocessing is performed and the DBSCAN algorithm is insensitive to noise data in our method, better segmentation results can be expected if the blood vessels can be further eliminated by effectively extracting vessels in the CT images [34]. Additionally, the image quality can be improved by suppressing noise artifacts in the low-dose CT images [35, 36].

For the lung CT image sequences of the three types of nodules, Table 8 further reveals the time complexity of the seven segmentation algorithms. The column “CT images” represents the average number of nodules contained in each CT sequence image. The average time consumption for each CT sequence using our method is 16.32 s; that is, the average segmentation time of a CT image is 1.36 s. In contrast, the segmentation speed of our method is faster than that of PCNN, KM, FCM, PSO-SGNN and FEGD. The average segmentation time of a single CT image is more than that of RG. However, considering the particularity of the lung image, more attention is focused on the precision of the segmentation in medicine. Therefore, the segmentation results of our method are more accurate and timely.

**Table 8:** Average execution time (s) of the five algorithms for all CT image sequences.

CT images	Criteria	Our method	RG	PCNN	KM	FCM	PSO-SGNN	FEGD
512*512*12	**Total**	16.32	8.28	83.88	17.88	19.92	27.96	188.64
	**Average**	1.36	0.69	6.99	1.49	1.66	2.33	15.72

512*512*12 represents the average number of nodules contained in each CT sequence image is 12, and the size of each CT image is 512×512.

## Conclusions

Accurate lung nodule segmentation is an important guarantee for late diagnosis. In this paper, to solve the problem of incomplete cavitary nodule segmentation, inaccurate juxta-vascular nodule segmentation and poor image sequence segmentation efficiency, a fast and accurate segmentation algorithm for lung nodules is proposed. To demonstrate the validity of the sequence segmentation algorithm, 1458 CT sequence images were selected for three types of nodules to perform a qualitative evaluation and quantitative comparison of all the segmentation results. The experimental results showed that compared with RG, PCNN, KM, FCM, PSO-SGNN and FEGD, the segmentation results of our method were much closer to the expert segmentation results, especially in terms of achieving accurate cavitary and juxta-vascular nodule segmentation. Those two types of nodule images are difficult to segment. Additionally, the mean PRI, GCE and VoI achieved using our method reached (0.9190, 0.0736, 2.2933) and (0.9126, 0.0833, 2.4658) for cavitary and juxta-vascular nodules, respectively; these values are clearly better than those achieved by other algorithms, reflecting the superiority of the segmentation effect of our method. Therefore, our method can efficiently and accurately segment lung nodule image sequences.

In the present study, we found that the target margins of ground-glass opacity lung nodules is fuzzy and will increase the difficulty associated with accurately segmenting the target. Therefore, the precise segmentation of such nodules will be our next goal.
